# Does retirement affect secondary preventive care use? Evidence from breast cancer screening

**DOI:** 10.1016/j.ehb.2021.101061

**Published:** 2021-12

**Authors:** Peter Eibich, Léontine Goldzahl

**Affiliations:** a Max Planck Institute for Demographic Research, Konrad-Zuse-Str. 1, 18057 Rostock, Germany; b Health Economics Research Centre, Nuffield Department of Population Health, University of Oxford, UK; c EDHEC Business School, 24 Avenue Gustave Delory, CS 50411, 59057 Roubaix Cedex 1, France

**Keywords:** Europe, Retirement, Health behavior, Instrumental variables, Preventive care, Breast cancer

## Abstract

This paper examines the causal impact of retirement on preventive care use by focusing on breast cancer screening. It contributes to a better understanding of the puzzling results in the literature reporting mixed effects on health care consumption at retirement. We use five waves of data from the Eurobarometer surveys conducted between 1996 and 2006, covering 25 different European countries. We address the endogeneity of retirement by using age thresholds for pension eligibility as instrumental variables in a bivariate probit model. We find that retirement reduces mammography use and other secondary preventive care use. Our results suggest that health status, income, and knowledge on cancer prevention and treatment contribute little to our understanding of the effects of retirement. Instead, our evidence suggests important effect heterogeneity based on the generosity of the social health insurance system and organized screening programs.

## Highlights

• Retirement reduces mammography use among European women.
• We also find reductions in other types of women’s secondary preventive care use.
• Changes in health, income and health beliefs do not seem to be relevant mechanisms.
• Effects occur mainly in countries without organized screening programs.
• Effects are larger in countries with less generous social health insurance.

## 1 Introduction

Population ageing is resulting in an increasing burden of non-communicable diseases for which age is a major risk factor, such as cardiovascular diseases, cancer or type 2 diabetes. For instance, the incidence of cancer in those over 65 is 10 times greater than in those younger than 65 and the cancer death rate is 16 times greater in patients over 65 compared to younger people during the early 2000s (Berger et al., 2006).

Many of these diseases are amenable to prevention. Changes in health behavior such as exercise or quitting smoking (i.e., primary prevention) can reduce the incidence of these diseases, while early detection (i.e., secondary prevention) can improve treatment outcomes and result in higher survival and fewer complications. Thus, preventive care could play a major role in maintaining the health of an ageing population.

Retirement is a major transition for this ageing population, and a growing part of the economic literature has examined the health effects of retirement (see Nishimura et al. (2018) and Rose (2020) for an overview). Several recent studies have focused on the effects of retirement on healthcare utilization with mixed results. Some studies report a decrease in healthcare use (Bíró and Elek, 2018, Eibich, 2015, Frimmel and Pruckner, 2020, Hallberg et al., 2015, Nielsen, 2019), whereas others find no effects (Hagen, 2018) or even an increase in healthcare use (Bíró, 2016, Lucifora and Vigani, 2018, Zhang et al., 2018).^1^ Health care use encompasses different types of care such as hospital care and doctor visits. Yet, even focusing on doctor visits yields mixed results. Eibich (2015), Nielsen (2019) -only for women-, Frimmel and Pruckner (2020), and Rose (2020) – only for women - find a decrease in doctor visits, while Lucifora and Vigani (2018) and Zhang et al. (2018) find an increase, and Rose (2020) no change among men. However, with few exceptions (Frimmel and Pruckner, 2020) these studies rely on general measures of healthcare use, such as visits to general practitioners or healthcare expenditures, which do not distinguish between curative and preventive healthcare, even though it appears plausible that there might be important differences between types of healthcare. For example, the theoretical model proposed by Galama et al. (2013) suggests that retirees reallocate expenditures away from healthcare investments and into consumption, because their retirement income is no longer dependent on their health status. Although the authors do not explicitly distinguish between curative and preventive care, it seems plausible that retirees reduce only expenditures on preventive care, because curative medical care provides immediate relief from symptoms. This is in line with (Cropper, 1977) who suggests that retirees reduce investments in preventive care due to the shorter payoff period.

Although a lot of attention has been devoted to the effect of retirement on primary prevention such as exercise (Celidoni and Rebba, 2017, Eibich, 2015, Insler, 2014, Kämpfen and Maurer, 2016, Motegi et al., 2016, Zantinge et al., 2014), only two other studies analyzed the effect of retirement on secondary prevention. Both Coe and Zamarro (2015) and Frimmel and Pruckner (2020) examine the effect of retirement on healthcare utilization and include screening participation as outcomes. Frimmel and Pruckner (2020) use Austrian register data and find a decrease in participation in health check-ups and prostate-specific antigen testing for men, but no changes in gynecological screening and mammography use for women. Coe and Zamarro (2015) examine cholesterol checks and prostate cancer screening using HRS data. They report no significant changes.^2^ Yet, the mechanisms behind these changes remain unclear. Decreases in healthcare utilization are typically attributed to the positive health effects of retirement (e.g., Frimmel and Pruckner, 2020; Hallberg et al., 2015), whereas increases in healthcare utilization are often explained by the decreasing opportunity costs of time investments (Lucifora and Vigani, 2018, Zhang et al., 2018). In addition, Frimmel and Pruckner (2020) examine heterogeneity by occupation and find that their results (for the screening outcomes) are primarily driven by blue-collar workers. Coe and Zamarro (2015) consider heterogeneity between health systems using SHARE data, and thus do not report estimates for their screening outcomes.

This paper extends the literature by examining the effect of retirement on secondary preventive care with a particular focus on breast cancer screening participation, since breast cancer is the most common cancer among women aged 50 and above. In this paper, we draw on five waves of repeated cross-section data from the Eurobarometer surveys collected between 1996 and 2006 and covering 25 EU countries. We use variation in state pension ages across countries as well as variation within countries over time to identify the causal effect of retirement. Our results suggest that retirement reduces mammography use, but also other secondary preventive care such as manual breast examinations and examinations of the ovaries. We also systematically investigate potential mechanisms, including some mechanisms that have not been considered previously, such as knowledge on breast cancer prevention and treatment, in addition to commonly discussed mechanisms by health status and income. We find that these potential mechanisms contribute little to our understanding of the negative effects of retirement.

Then, we examine whether the effect of retirement on breast cancer screening participation differs between countries with and without national organized screening programs, with high or low social health insurance (SHI) coverage, and across education levels. We find that the impact of retirement is more pronounced in countries without screening programs, whereas it is smaller and insignificant in countries with organized screening programs. It is also larger in countries with less generous social health insurance coverage, and among individuals with medium and high education.

Our study makes contributions along several dimensions. First of all, we contribute to the literature on retirement and healthcare utilization by focusing on one specific type of healthcare: secondary preventive care. Evidence from existing empirical studies on retirement and healthcare use is mixed. Thus, it appears necessary to distinguish between different types of healthcare, as theoretical considerations suggest that the mechanisms linking retirement and healthcare use might differ between curative and preventive care. Moreover, this is one of the first studies to provide comprehensive evidence of the effect of retirement on secondary preventive care among women, and especially for breast cancer screening. While Frimmel and Pruckner (2020) also considered mammography use in a single country, we also investigate whether the effect changes with the extent of social health insurance coverage, if an organized screening program is in place, and provide detailed evidence on other types of secondary prevention for women across 25 different countries. Second, we add to the existing literature by investigating a new mechanism - changes in knowledge on breast cancer prevention and treatment at retirement. Third, the cross-country nature of our data, and the gradual implementation of screening programs in Europe allows us to study effect heterogeneity by institutional settings such as the existence of a screening program.

The next section reviews the theoretical background and the existing empirical literature. Section 3 presents the context and the data, while Section 4 describes the methods. Section 5 presents our results and the mechanisms through which retirement affect mammography utilization are depicted in Section 6. Section 7 provides insights into effect heterogeneity. The final section discusses the results and concludes.

## 2 Theoretical considerations and relevant empirical literature

In the following, we will discuss theoretical models and empirical studies that may explain how retirement affects secondary preventive care use. It is important, in particular for empirical findings, to distinguish between mechanisms and effect heterogeneity. A mechanism implies that the causal effect of retirement on secondary preventive care use operates (at least partly) through a mediator, which itself is causally affected by retirement. Effect heterogeneity implies that the causal effect of retirement on secondary preventive care use differs between certain groups. The modifier used to distinguish these groups does not necessarily need to be affected by retirement itself. Effect heterogeneity therefore does not clearly explain how retirement affects secondary preventive care use, but it may be suggestive of certain mechanisms.

### 2.1 Potential mechanisms behind the effect of retirement on breast cancer screening

Previous empirical studies have primarily considered time constraints as the main mechanism linking retirement to changes in primary prevention. Theoretical considerations suggest that income, health, as well as knowledge on prevention and treatment could also explain the relationship between retirement and secondary preventive care use.

Retirement typically leads to a reduction in income. Healthcare might therefore be less accessible after retirement if retirees have less income to pay for co-payments, out-of-pocket expenditures or transportation costs. Galama et al. (2013) suggest another interesting theoretical mechanism based on Grossman’s health capital model (Grossman, 1972). Their model predicts that healthcare consumption should decrease after retirement. Post-retirement income is independent of health. While health still has a direct effect on utility, it ceases to affect utility indirectly through consumption, and therefore retirees are expected to reallocate resources from health investments into higher consumption. According to both of these income-driven mechanisms, retirement may lead to a decrease in mammography use.

There is a large literature investigating the health effects of retirement (Bloemen et al., 2017, Bozio et al., 2021, Celidoni and Rebba, 2017, Coe and Zamarro, 2011, De Grip et al., 2012, Eibich, 2015, Grøtting and Lillebø, 2020, Hallberg et al., 2015, Insler, 2014, Nishimura et al., 2018), with studies reporting contrasting findings even for similar health outcomes and institutional contexts (see, e.g., Rose, 2020 and Behncke, 2012 for England; or Dave et al., 2008 and Insler, 2014 for the U.S.).^3^ At the same time, several studies find that health is positively associated with participation in breast cancer screening (Bouckaert and Schokkaert, 2016, Carrieri and Wuebker, 2016, Courtney-Long et al., 2011, Gandhi et al., 2015, Guilcher et al., 2014, Jensen et al., 2015, Wu, 2003). Taken together, these findings suggest that retirement could affect mammography use through its effect on health but the sign is ambiguous – if retirement improves health, then mammography use might be expected to increase, whereas a negative effect of retirement on health would imply a decrease.

Transitions out of the labor force at older ages may induce changes in individual’s social networks in terms of both size and composition of the network. Evidence from the US shows that retirement reduces the size and density of social networks (Patacchini and Engelhardt, 2016). Evidence from Europe is mixed. Fletcher (2014) finds that retirement had little impact on social network size while Börsch-Supan and Schuth’s (2013) results show that retirement negatively impacts cognitive health through a reduction of the social network. These findings also suggest that changes in social networks and cognitive functioning occur shortly after retirement (Bonsang et al., 2012, Patacchini and Engelhardt, 2016). Social networks can influence individuals’ behavior by circulating information on the effectiveness of recommended health behavior (Berkman and Glass, 2000). Knowledge about breast cancer screening has been shown to be a key determinant of mammography utilization (Dündar et al., 2006, Ferrat et al., 2013, Grunfeld et al., 2002, Lagerlund et al., 2000, Rakhshkhorshid et al., 2018). In addition, evidence shows that social network measured as the number of contacts with family and friends as well as emotional social support (i.e., having someone to discuss personal concerns with) have a positive influence on mammography participation (Jensen et al., 2015). Taken together, these findings suggest that retirement may affect mammography through changes in breast cancer prevention and treatment knowledge since social networks change at retirement. If retirement reduces the size of social networks, it diminishes the influx of new and relevant information, leading to a reduction in mammography use. Retirement can also shift the composition of the network from (younger) colleagues towards older friends and peers, thus leading to more pessimistic views of breast cancer prevention and treatment, which can have either a positive or negative effect on mammography use.

Previous studies show that retirement improves health behavior, and in particular those behaviors that require a time investment, e.g., exercise (Eibich, 2015, Insler, 2014, Kämpfen and Maurer, 2016, Motegi et al., 2016), sleep duration (Eibich, 2015, Motegi et al., 2016) and GP or specialist visits (Lucifora and Vigani, 2018). This suggests that retirement positively affects primary and secondary preventions through lower time constraints, since retirees have more leisure time available, which decreases the time costs of health investments.

### 2.2 Heterogeneity of the effect of retirement on breast cancer screening

Screening programs aim to reduce access barriers to healthcare by offering screening free of charge. Programs also reduce the time costs by inviting women directly for screening without the need to obtain a referral from their GP first. Lastly, the information provided alongside an invitation for screening in an organized program could affect women’s knowledge on their cancer risk and of the benefit of screening (Martínez-Alonso et al., 2017, Woloshin et al., 2012). Therefore, it appears plausible that the effect of retirement on mammography use might differ between countries with a screening program and those without a program.

The extent of social health insurance coverage in a country affects healthcare access through how much out of pocket expenses are not covered by social insurance. The cost of a mammogram is considerable relative to other routine preventive examinations. As an illustration, the costs are between 40 and 66 euros in Spain and France and up to 110 euros in Germany.^4^ A related potential mechanism are changes in complementary health insurance coverage at retirement. For example, in the US, individuals become eligible for Medicare coverage at 65. In contrast, European countries typically have a social health insurance system covering both workers and retirees. However, employees might benefit from employer-sponsored complementary health insurance, workplace-based prevention programs or incentives to participate in existing prevention programs. Thus, the extent of health insurance coverage might affect the financial barrier to obtaining a mammogram, such that better coverage leads to increased mammography use (Bitler and Carpenter, 2016). Retirement might reduce the likelihood to use secondary preventive care if retirees are not covered by such schemes. This is unfortunately an untestable mechanism, because private health insurance is not reported in our data.

The literature shows an education gradient in preventive care utilization in Europe (Jusot et al., 2012). One rationale behind this stylized fact is derived in Grossman (2000). According to the allocative efficiency assumption, the more educated are assumed to be more able to select inputs to produce health than the less educated (Grossman, 2000). Career paths and occupation also differ with educational level resulting in variation in exposure to physical strain and related health deterioration. We expect that the effect of retirement on breast cancer screening depends on educational attainment.

## 3 Contextual setting and data

### 3.1 Contextual setting

#### 3.1.1 Breast cancer screening in Europe

We focus on breast cancer screening for three reasons: First, breast cancer is the most common type of cancer in women worldwide (WHO, 2017a). The incidence of breast cancer is estimated to be 494,176 in Europe and 361,608 in the 28 countries of the European Union in 2012 (WHO, 2017b). Thus, 1 in 8 women in the EU-28 will develop breast cancer before the age of 85 (Ferlay et al., 2007). In 2015, roughly 91,585 women in the 28 countries of the European Union and 142,979 women in Europe died of breast cancer (WHO, 2017b). Second, breast cancer is most common in women above 50 years of age (WHO, 2017a), i.e., those women close to retirement or already retired. Third and last, if detected early, breast cancer is highly treatable with very high rates of survival. Survival rates depend crucially on the stage at which breast cancer is detected. Early screening increases the probability to detect a cancer at a more local stage, hence improving survival. The most common method for early detection of breast cancer is mammography - low dose X-ray imaging of the breasts. Based on evidence from clinical trials indicating that screening mammography reduces mortality by detecting tumors at an earlier stage (Marmot et al., 2013) expert organizations (World Health Organization's International Agency for Research on Cancer and the American Cancer Society) recommend regular, biennial screening mammograms starting at age 50 (Perry et al., 2008). Nearly every European country has now established a national breast cancer screening program (Altobelli and Lattanzi, 2014). The screening programs in Finland (1989), the UK (1995) and Sweden (1996) were introduced before the period covered in our empirical analysis (1996–2006, see Table A.1 in the Online Appendix). Between 1996 and 2006, national breast cancer screening programs were introduced in seven further countries, three of which are observed both before and after the introduction of the programs. All other countries, except Greece, have introduced a national screening program since 2006. Most countries provide free mammography for women aged over 50 years until 69 or 74 years old every 2 to 3 years, although a few countries offer screening from as early as 40 years. Such programs have successfully increased mammography use in Europe (Buchmueller and Goldzahl, 2018, Carrieri and Wuebker, 2016, Pletscher, 2017) and decreased mortality in the US and in the UK (Leive and Stratmann, 2015). 5 Breast cancer screening includes a mammography and a clinical exam performed by a radiologist. European breast cancer screening programs provide free breast cancer screening, with no upfront fees and don’t require a prescription. These programs typically send an invitation letter to eligible women to inform them about their eligibility, which also contains detailed information about the age range, screening interval and breast cancer screening procedure. The European guidelines (Perry et al., 2008) indicate that at least 70% of women invited should attend breast cancer screening.^6^ In European countries, non-eligible women (either because they are outside the eligibility age range or they live in a country without a program) have access to breast cancer screening upon prescription provided by GPs or gynecologists. While breast cancer screening is often covered by national health insurance, coverage varies by country.

In some countries with a screening program (France, Switzerland, Luxemburg, Austria or Belgium), women within the age range can be screened in the program as well as outside the program (“opportunistic screening”). Screening outside of the program might incur out-of-pocket expenditures.

#### 3.1.2 Retirement eligibility

The pension systems of most European countries involve both an Official Retirement Age (ORA) and an Early Retirement Age (ERA). The ORA represents the age at which all workers can claim a full old age pension, while the ERA offers specific subgroups the opportunity to retire at an earlier age. As pension eligibility might involve other criteria,^7^ our data does not allow us to ascertain pension eligibility on the individual level. Therefore, for the purpose of this study we define the ORA as the age at which all women are able to claim a pension, while the ERA is defined as the earliest age at which a woman might be eligible for a pension.

We use data on the ORA and ERA for each survey year and country from the database of the Mutual Information System on Social Protection (MISSOC, 2017). There is considerable variation in the ERA and ORA, both between countries as well as within countries over time. Further information on the pension ages used in this study is provided in Table A.1 in the Online Appendix.

### 3.2 Data

#### 3.2.1 Eurobarometer

The analysis is based on data from the Eurobarometer. The Eurobarometer is a series of cross-sectional surveys conducted on behalf of the European Commission. The surveys are conducted several times per year and include individuals from all current member states of the European Union. The surveys cover a range of different topics, which are based on current information needs of the European Commission and the European Parliament. Data from the Eurobarometer surveys are available to the scientific community via the Eurobarometer Data Service at GESIS (GESIS, 2017). The two main advantages of this survey compared to similar ones (such as the Survey of Health, Ageing and Retirement in Europe) is the availability of questions on preventive care utilization as well as on breast cancer knowledge in several waves. In addition, the cross-country nature of the survey enables us to investigate the heterogeneity of the effect of retirement by institutional settings such as the existence of a screening program.

Table A.2 in the Appendix summarizes the number of observations per country and year in our sample. For the years 1996, 1997, 1998 and 2003, the sample is restricted to women living in the EU-15 countries. In 2006, the 10 accession countries are included in our sample. On average, the sample includes 212 women per country and year, with a range of 70 (Luxembourg) to 525 (Germany). Unfortunately, response rates for the Eurobarometer are not routinely published. For the 1996 wave (EB44.3) included in our analysis, an average response rate of 52% is reported for the basic sample (Gallie, 1996), however, the response rates vary widely across countries. The Eurobarometer data includes post-stratification survey weights (derived from the national Labour Force Surveys) to address non-response, which we use in a robustness check (see Table A.5 in the Online Appendix).

#### 3.2.2 Outcomes

For this paper, we use a set of questions on women’s preventive healthcare use that was included five times between 1996 and 2006.^8^ These questions asked whether the woman had any of the following medical check-ups in the past 12 months: a mammography, a manual breast examination, a pap smear test (i.e., cervical cancer screening), an examination of the ovaries, a test for osteoporosis, or any other gynecological examination. Our focus is on mammography, since manual breast examinations are less effective at detecting early-stage breast cancer. Ovarian and cervical cancers are less common than breast cancer for the age group of interest. Furthermore, very few countries had cervical cancer screening programs (and none had programs for ovarian cancer) at the time of the survey and if they had one, the maximum age for eligibility would be 65 years old. Likewise, osteoporosis tests are rarely conducted and there are no screening programs for osteoporosis.

#### 3.2.3 Retirement definition

We define women as retired if their self-reported occupational status is either “retired”, “permanently sick or injured”, or “homemaker”, since these women are unlikely to re-enter the labor market. In contrast, unemployed individuals are not considered to be retired, since they are looking for work and might re-enter the labor market. Unfortunately, we cannot distinguish between “retired” and “permanently sick or injured” in the Eurobarometer data, since these two options are in the same category (self-reported occupation as “retired or unable to work through illness”). Conflating retirement and permanent sickness or injury introduces a measurement error, which is, however, resolved through our instrumental variables (IV) approach (see Section 4).

We conduct robustness checks for including or excluding homemakers and unemployed women as retired. To address selective labor market participation, we exclude women from the analysis who reported they had never done paid work.^9^

We complement the survey data with information on state pension ages and existence and coverage of screening programs as discussed in the previous section (available in the Appendix in Table A.1.).

#### 3.2.4 Mechanisms

For the analysis of potential mechanisms, we investigate women’s income, health status and knowledge concerning breast cancer prevention and treatment.

Income is available in 1996, 1997, 1998, 2003, and approximately 25–33% of the sample declined to answer this question each year. Unfortunately, this implies that the sample of women who reported their household’s income may be strongly selective. Respondents are provided with country-specific income categories and are asked to indicate in which category their monthly gross household income is. This categorical income variable is then further collapsed into country-specific quartiles. For this study, we then use a binary indicator that measures whether the respondents self-reported monthly gross household income is above or below their country’s median income.

Self-assessed health status is only available for years 1996, 1998, and 2006, but in contrast to income there are very few missing responses. Respondents are asked whether they would rate their health over the past 12 months as “very bad”, “bad”, “fair”, “good”, or “very good”. We collapse these responses into a binary indicator, which takes on the value of 1 if respondents rated their health as “good”, “very good”, or “fair”.

In 1997 and 1998 only, women were asked whether they thought the following statements were true or false:

- “The sooner a cancer is detected, the better it can be treated.”
- “A mammography will detect signs of breast cancer.”
- “There are effective treatments for breast cancer.”
- “In most cases, you can be cured of breast cancer if it is detected early enough.”
- “Removal of the breast is the only way to be cured of breast cancer.”.

Women were also asked whether they personally thought that cancer can be prevented.^10^

Using these six items, we construct an index measuring the stock of health knowledge for each woman in line with Eibich and Goldzahl (2020), Kenkel (1990)’s and Hsieh and Lin (1997)’s methods. We assign a score of 1 if women stated that the statement is “true”, and 0 if they responded “don’t know” or “false”. The item on breast removal was reverse coded. Every correct answer adds 1 point to the score. The resulting index varies between 0 and 6, and can be interpreted as the number of correct answers.

It is important to note that the sample for the analysis of these mechanisms is considerably smaller than the sample for our main analysis, and therefore the findings on these mechanisms should be interpreted with caution.

#### 3.2.5 Heterogeneity

First, the staggered implementation of breast cancer screening programs in Europe enables us to construct a dummy variable for program existence in each year and each country. Table A.1 provides the year in which nationwide coverage of breast cancer screening programs was achieved.

Second, we examine whether the negative effect of retirement on mammography use varies with social health insurance (SHI) coverage. All European countries in our study have a SHI system that covers a large share of healthcare expenditures for both working and retired individuals. In countries where all expenditures are covered by SHI, retirement should not affect healthcare access, since complementary health insurance plays no role in providing access to healthcare. In contrast, we would expect that the effect of retirement on secondary preventive care use is more pronounced in countries with a lower share of costs covered by SHI, since complementary health insurance is (relatively) more important in providing access to secondary preventive care. Hence, we investigate heterogeneity across institutional settings based on differences in coverage of SHI. We use OECD data on healthcare expenditures (OECD, 2017) by year covering 23 out of the 25 countries in our analysis.^11^ We construct an indicator for “SHI coverage”, which we define as the ratio of government and compulsory healthcare expenditures to total healthcare expenditures for each year of the survey. A value of 1 would indicate that all healthcare expenditures are covered by the SHI system (e.g., tax financed as in the UK, or funded by compulsory health insurance schemes as in Germany), while a value of 0 indicates that all healthcare expenditures are voluntary (i.e., either out-of-pocket payments or covered by private health insurance schemes). In our sample, the social health insurance indicator ranges from 0.49 to 0.92. We create a binary variable, which divides our sample into a group of countries with relatively high SHI coverage and a group of countries with lower SHI coverage.

Third, we use educational attainment information available in each wave of the Eurobarometer to observe heterogeneity by educational levels. Education categories are based on age at which the respondent finished full-time education, with “at 15 or younger” corresponding to a low level of education, “between 16 and 19” to medium, and “20 years or older” to high education.

#### 3.2.6 Sample description

We restrict our working sample to women aged between 45 and 75 to ensure that for every country we include observations below the ERA and above the ORA.^12^ In addition, in some countries screening programs invite women from age 40 onwards, and almost all screening programs offer screening up to the age of 69 or 74. Table A.3 in the Appendix shows summary statistics for the working sample.^13^ We note that about 35% of the women had a mammography in the past 12 months, while 39% had a manual breast examination (i.e., either a self-examination or an examination by a clinician). Agreement to the statements on early detection, prevention and treatment of breast cancer was generally very high, with the exception of “Removal of the breast is the only way to be cured of breast cancer”. However, it is worth noting that 40% of the women thought that cancer cannot be prevented. 35% of the women lived in a country with an organized screening program in the year of the survey.

## 4 Methods

### 4.1 Instrumental variables and bivariate probit estimation

**Fig. 1:**
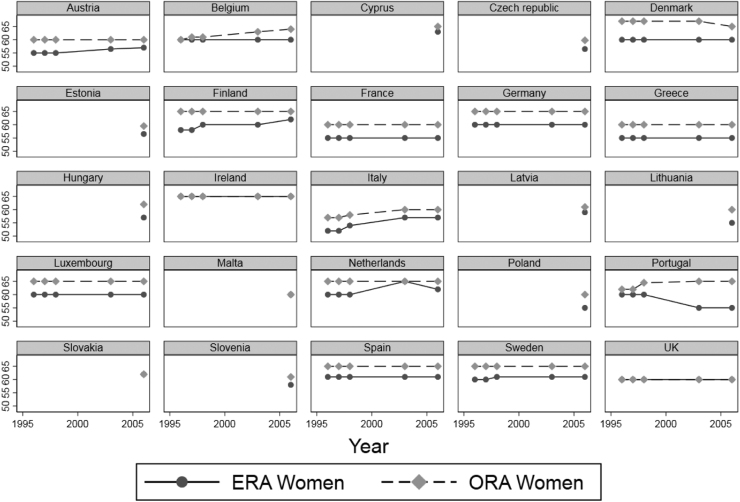
Variation in state pension ages.

Source: Social protection in the member states of the European Union, MISSOC (1994, 1997, 1998, 2003), Celidoni and Rebba (2017), Euwals et al. (2010), Jousten et al. (2010), Mazzonna and Peracchi (2014) and Staubli and Zweimüller (2013).

In line with previous studies on retirement and health (see, e.g., Coe and Zamarro, 2011; Godard, 2016; Mazzonna and Peracchi, 2012), we exploit age thresholds for pension eligibility as a source of exogenous variation in retirement status in an instrumental variables estimation. Fig. 1 shows the variation in state pension ages for each country in our sample over the study period (based on Table A.1 in the Online Appendix). We note that there is considerable variation between countries. For example, throughout the study period, women in France could retire early at 55, while in Germany the early retirement age was 60. Similarly, there is considerable variation within countries over time. While several countries raised their state pension ages (e.g., Italy increased the ERA from 52 in 1996–57 in 2006), there are also a few countries that lowered the ERA or ORA, respectively, e.g., Denmark or Portugal.

These thresholds provide a financial incentive for individuals to postpone retirement until they have reached a certain age, since they are not able to draw upon their state pension beforehand. We therefore expect that the likelihood of retirement increases strongly at these state pension ages. Fig. A.1 in the Online Appendix plots the share of retired women in the sample against the time until women reach their country’s respective state pension age.^14^ The figure shows an increase in the retirement probability of about 20% points at the ERA, and a smaller increase of about 10% points at the ORA. This suggests that ERA and ORA are relevant predictors of retirement.

Given that the state pension ages vary between countries as well as within countries over time, they should not be correlated with unobserved confounders, such as health status. Fig. A.3 in the Online Appendix shows that we do not observe any discontinuous changes at the age thresholds for state pension eligibility for marital status and household size.^15^ We interpret this as suggestive evidence that the assignment of the instrument (conditional on age) can indeed be considered random, i.e., women above and below the age threshold are comparable in their characteristics other than retirement. Thus, we can use these state pension ages as instrumental variables for retirement.

Moreover, the instrumental variable approach can also address potential measurement error in our retirement variable, which might stem from conflating retirement status and permanent sickness or injury. Women’s likelihood to be permanently sick or injured (and thus coded as “retired” in our data) should not change at the age thresholds for state pension eligibility, and therefore permanently sick or injured women do not contribute to the identification of our IV estimate.

We estimate bivariate probit models with binary indicators for whether women have exceeded the relevant ERA or ORA in their countries as instruments for retirement. All our outcome measures are binary variables, and the bivariate probit model allows us to take the limited support of these variables into account. Although the linear probability model (LPM) is often assumed to provide very similar results to models for binary dependent variables, it has been shown that the LPM is potentially biased and inconsistent (Horrace and Oaxaca, 2006). In simulations and empirical applications the results from 2SLS models and nonlinear IV estimators can diverge substantially (Basu et al., 2018, Bhattacharya et al., 2005, Chiburis et al., 2012, Terza et al., 2008), although it is not clear whether linear or nonlinear IV estimators should generally be preferred. For this paper, we also estimated linear probability two-stage least squares models and two-stage residual inclusion (2SRI) models with logistic regressions in the first- and second-stage. The results (see Table A.7 in the Online Appendix) are qualitatively similar to our findings from the bivariate probit model presented here. However, the point estimates from the bivariate probit model are smaller than those from the 2SRI logit-logit model, and considerably smaller than the linear 2SLS estimates. Therefore, the bivariate probit model is our preferred specification, as it represents a more conservative approach.

We control for a quadratic age trend (measured in years), four binary categories of education (finished full-time education at *(i)* 15 years or younger, *(ii)* between 16 and 19, *(iii)* at 20 years or older, *(iv)* still studying), and country- as well as year-fixed effects. The inclusion of additional covariates and fixed effects is not necessary for causal identification in IV models. IV estimation addresses all potential sources of bias as long as the necessary assumptions are met.^16^ Here, we include controls for education and country- as well as -year-fixed effects to address the potential correlation between differences in pension eligibility ages across countries and education (Bingley and Martinello, 2013).

The resulting model can be written as follows:

(1a) $$\begin{array}{c} retired_{i}=1\{\alpha +\beta_{1}Age_{i}+\beta_{2}Age_{i}^{2}+\tau_{1}ERA_{i}+\tau_{2}ORA_{i}\\ +\sum_{j=2}^{4}\delta_{j}Educ_{j,i}+\gamma_{c}+\delta_{t}+\varepsilon_{i}>0\} \end{array}$$

(1b) $$\begin{array}{c} Screen_{i}=1\{\mu +\theta_{1}Age_{i}+\theta_{2}Age_{i}^{2}+\pi Retired_{i}\\ +\sum_{j=2}^{4}\rho_{j}Educ_{j,i}+\gamma_{c}+\delta_{t}+\nu_{i}>0\} \end{array}$$

In the first stage of the model, we regress retirement of observation *i* ($\mathrm{retire}d_{i}$) on binary indicators of whether women *i* is above or below the ERA and ORA in country *c* and year *t* ($\mathrm{ER}A_{i}$ and $\mathrm{OR}A_{i}$), while controlling for a quadratic age trend, four binary indicators of education and country- as well as year-fixed effects ($\gamma_{c}$ and $\delta_{t}$, respectively). In the second stage, we regress mammography use on the instrumented retirement status. The bivariate probit model assumes that the error terms $\epsilon_{i}$ and $\nu_{i}$ follow a bivariate normal distribution, and the correlation between these error terms is explicitly estimated. Identification in this model comes from both the exclusion restriction on the instrumental variables as well as the functional form assumptions on the joint distribution of the error terms.^17^ Following Chiburis et al. (2012), we use standard errors derived from 200 bootstrap replications.^18^

### 4.2 Sensitivity analysis and robustness checks

We systematically explore the sensitivity of our findings in a specification curve analysis (Christensen and Miguel, 2018, Simonsohn et al., 2020). This approach requires that researchers specify a priori plausible alternatives for the modeling choices made in their analysis. The set of all a priori plausible specifications is then defined by combining these alternative modeling choices. In a specification curve analysis, all specifications within this set are then estimated. The results can then be visualized to identify which combinations of modeling choices have a major impact on the results. For this study, we consider the sensitivity of our findings regarding *(i)* the age polynomial (linear, country-specific quadratic, quadratic, cubic), *(ii)* the included age range (45–75 or only within screening program age range), *(iii)* the definition of retirement status (whether homemakers and unemployed women are considered retired), and *(iv)* the exclusion of the observations within the first year after reaching the ERA/ORA. We focus on the age polynomial and the included age range, because the validity of state pension ages as an instrument only holds conditional on a correctly specified age trend. The definition of retirement has received considerable attention in the literature on the health effects of retirement (Nishimura et al., 2018), but a clear consensus has not yet emerged. Finally, we consider “donut”-specifications, which exclude observations within the first year after reaching the ERA or ORA, because our outcomes measure secondary preventive care use in the last 12 months. Other methodological choices are less likely to influence our findings (e.g., the inclusion of exogenous covariates should not affect the estimates in an IV model) or are rarely considered in the literature on retirement and health (e.g., the use of survey weights). Therefore, we focus on the four modeling choices discussed above, which result in 48 different specifications, which we estimate and visualize.

In addition, we also conduct several robustness checks, in which we examine the robustness of our results with respect to *(i)* the inclusion of survey weights, *(ii)* limiting our sample to include only EU-15 countries, *(iii)* considering only women within their respective country’s screening program target age range, *(iv)* restricting our sample to countries which did not introduce a screening program between survey waves, *(v)* the three different retirement definitions noted above, *(vi)* excluding observations within the first 12 months of their country’s ERA or ORA, and *(vii)* a specification which includes control variables for household size and marital status. We also estimate models using placebo state pension ages as a falsification exercise. For each observation in our sample we generate a placebo state pension age by drawing a random number between 50 and 70 from a uniform distribution, and we construct our instruments based on these random numbers. Finally, we consider the robustness of our results to functional form assumptions by re-estimating our IV models using linear 2SLS as well as a logit-logit 2SRI specification.

### 4.3 Mediation analysis

We follow the approach by Tubeuf et al. (2012) (which is based on work by Karlson et al., 2012) to examine the potential mechanisms behind the impact of retirement on secondary preventive care use. The mediation analysis aims at measuring the proportion of the effect of retirement on breast cancer screening that operates indirectly through variation in a mediating variable. To examine the relevance of a mediator $m_{i}$, we first estimate a linear 2SLS model to estimate the impact of retirement on the mediator:

(2b) $$m_{i}=\zeta_{1}+\zeta_{2}\mathrm{Ag}e_{i}+\zeta_{3}\mathrm{Ag}e_{i}^{2}+\kappa \mathrm{Retire}d_{i}+\sum_{j=2}^{4}\chi_{j}\mathrm{Edu}c_{j,i}+\gamma_{c}^{m}+\delta_{t}^{m}+\omega_{i}$$

with the corresponding first stage:

(2a) $$\mathrm{retire}d_{i}=\eta_{1}+\eta_{2}\mathrm{Ag}e_{i}+\eta_{3}\mathrm{Ag}e_{i}^{2}+\lambda_{1}\mathrm{ER}A_{i}+\lambda_{2}\mathrm{OR}A_{i}+\sum_{j=2}^{4}\psi_{j}\mathrm{Edu}c_{j,i}+\gamma_{c}^{r}+\delta_{t}^{r}+\varepsilon_{i}.$$

Our bivariate probit model can be rewritten as follows to account for the mediator:

(3a) $$\begin{array}{c} retired_{i}=1\{\alpha +\beta_{1}Age_{i}+\beta_{2}Age_{i}^{2}+\tau_{1}ERA_{i}+\tau_{2}ORA_{i}\\ +\sum_{j=2}^{4}\phi_{j}Educ_{j,i}+\gamma_{c}^{r}+\delta_{t}^{r}+\varepsilon_{i}>0\} \end{array}$$

(3b) $$Screen_{i}=1\left\{\tilde{\mu }+{\tilde{\theta }}_{1}Age_{i}+{\tilde{\theta }}_{2}Age_{i}^{2}+\tilde{\pi }Retired_{i}+\xi m_{i}+\sum_{j=2}^{4}{\tilde{\rho }}_{j}Educ_{j,i}+{\tilde{\gamma }}_{c}^{s}+{\tilde{\delta }}_{t}^{s}+{\tilde{\nu }}_{i}>0\right\}$$

However, estimating the model in Eq. (3b) and comparing the coefficients to those obtained from Eq. (1b) is problematic, because of the rescaling of the variance of the latent variable across models (Karlson et al., 2012). However, we can rewrite Eq. (3b) by substituting $m_{i}$ using Eq. (2b):

(4) $$\begin{array}{c} Screen_{i}=1\{\overset{\sim }{\mu }+{\overset{\sim }{\theta }}_{1}Age_{i}+{\overset{\sim }{\theta }}_{2}Age_{i}^{2}+\overset{\sim }{\pi }Retired_{i}\\ \overset{m_{i}}{\overset{⏞}{\begin{array}{c} +\xi \left(\zeta_{1}+\zeta_{2}Age_{i}+\zeta_{3}Age_{i}^{2}+\kappa Retired_{i}+\overset{4}{\underset{j=1}{\sum }}\chi_{j}Educ_{j,i}+{\gamma^{m}}_{c}+\delta_{t}+\omega_{i}\right)\\ +\overset{4}{\underset{j=2}{\sum }}{\overset{\sim }{\rho }}_{j}Educ_{j,i}+{\overset{\sim }{\gamma }}_{c}+{\overset{\sim }{\delta }}_{t}+{\overset{\sim }{\nu }}_{i}>0\}. \end{array}}} \end{array}$$

We can then rewrite Eq. (4) as:

(5) $$\begin{array}{c} \mathrm{Scree}n_{i}=1\{(\overset{\sim }{\mu }+\xi \zeta_{1})+({\overset{\sim }{\theta }}_{1}+\xi \zeta_{2})\mathrm{Ag}e_{i}+({\overset{\sim }{\theta }}_{2}+\xi \zeta_{3})\mathrm{Ag}e_{i}^{2}+(\overset{\sim }{\pi }+\xi \kappa )\mathrm{Retire}d_{i}+\xi \omega_{i}\\ +\sum_{j=1}^{4}\left({\overset{\sim }{\rho }}_{j}+\xi \chi_{j}\right)\mathrm{Edu}c_{j,i}+\left({\overset{\sim }{\gamma }}_{c}^{s}+\xi \gamma_{c}^{m}\right)+\left({\overset{\sim }{\delta }}_{t}^{s}+\xi \delta_{t}^{m}\right)+{\overset{\sim }{\nu }}_{i}>0\}\; \end{array}$$

If $\omega_{i}$ in Eq. (5) is unobserved, then $\xi \omega_{i}$ becomes part of the error term, in which case Eq. (5) is identical to Eq. (1b), i.e., $\nu_{i}={\tilde{\nu }}_{i}+\xi \omega_{i};\mu =\tilde{\mu }+\xi \zeta_{1};\pi =\tilde{\pi }+\xi \kappa$ etc. This means that in the presence of an unobserved mediator, the coefficients in Eq. (1b) capture both the direct effect of the respective variables on the outcome as well as the indirect effect operating through the mediator. In particular, the direct effect of retirement on secondary preventive care use is given by $\tilde{\pi }$. The indirect effect of retirement operating through the mediator *m* is given by ξκ, and the total effect of retirement on secondary preventive care use can be derived as $\pi =(\tilde{\pi }+\kappa \xi$). To derive estimates of these quantities, we first obtain the predicted residuals ($\hat{\omega_{i}}$) from the second stage of the auxiliary model (2b) and estimate Eq. (5) using the following, modified bivariate probit model:

(6a) $$retired_{i}=1\{\alpha +\beta_{1}Age_{i}+\beta_{2}Age_{i}^{2}+\tau_{1}ERA_{i}+\tau_{2}ORA_{i}+\sum_{j=2}^{4}\phi_{j}Educ_{j,i}+\gamma_{c}+\delta_{t}+\varepsilon_{i}>0\}$$

(6b) $$Screen_{i}=1\left\{\mu +\theta_{1}Age_{i}+\theta_{2}Age_{i}^{2}+\pi Retired_{i}+\xi {\hat{\omega }}_{i}+\sum_{j=2}^{4}\rho_{j}Educ_{j,i}+\gamma_{c}+\delta_{t}+\nu_{i}>0\right\}$$

From Eq. (6b), we can identify the total effect as $\pi =\tilde{\pi }+\xi \kappa$. Using the auxiliary regression in Eq. (2b) and the estimated coefficients from Eq. (6b), the indirect effect is given as ξκ, and from these two quantities we can derive the direct effect as $\tilde{\pi }=\pi -\xi \kappa$. We perform 200 bootstrap replications to derive standard errors around these quantities.

### 4.4 Heterogeneity analysis

We explore the heterogeneity of the effect of retirement on breast cancer screening across program existence, social health insurance coverage, and education levels. For each of these modifiers, we stratified the sample into relevant categories and reproduce our main analysis using the bivariate probit model for each category.^19^

We note that differences in the effects of retirement between these groups cannot be interpreted as causal without further, restrictive assumptions. For instance, there could be potential systematic differences in age-specific breast cancer incidence or mortality between countries that adopt a program and those which don’t. In that case, women living in countries with higher risk of breast cancer, which hence introduced a program, would get screened more often. Any difference that we would observe would be confounded by differences in age-specific incidence and mortality rates. The epidemiological literature suggests that this is not the case as observed differences in incidence trends by countries are due to the country-specific age structure (Bray et al., 2004). Furthermore, Althuis et al. (2005) use local registry data to compute incidence rates of the years 1993–1997, and national data for mortality rates. Although they don’t cover all European countries, there do not seem to be large differences in incidence and mortality rates between countries that had a program (the UK, Finland and Sweden) and those which did not (Italy, Spain and Denmark) at the time of the early survey waves. In Fig. A.5 in the Appendix, we plot age-adjusted mortality rates for breast cancer over time from the Global Burden of Disease database (Global Burden of Disease Collective Network, 2021). The figure suggests that there is no systematic correlation between the introduction of breast cancer screening programs and the age-adjusted mortality rates. However, even if the introduction of breast cancer screening programs is not related to the incidence and mortality from breast cancer, it is still possible that there might be other differences between countries that introduced breast cancer screening programs earlier and those that introduced their programs later (i.e., after 2006).

## 5 Results

Table 1 shows the estimated effect of our instruments on retirement status (i.e., the first stage) and on mammography use (i.e., the reduced form). First, we note that both instruments are significant predictors of retirement status – being above the ERA increases the retirement probability by about 11% points, while being above the ORA increases the retirement probability by 6% points. These estimates are in line with our expectations based on a visual inspection of the data (Fig. A.1 in the Online Appendix).

**Table 1:** First-stage regression and reduced form estimates.

	Outcome: Retired	Outcome: Mammography use in the past 12 months
Above ERA	0.107***	-0.044***
	(0.011)	(0.013)
Above ORA	0.055***	-0.039***
	(0.013)	(0.015)
N	17,875	17,875

*Sources*: Eurobarometer, own calculations. Estimates are average marginal effects from probit regression models. The models include a quadratic age trend, education and country- and year- fixed effects. The sample includes women aged 45–75. Standard errors shown in parentheses are based on 200 bootstrap replications. ***p < 0.01; **p < 0.05; *p < 0.1.

The reduced form estimates (in line with the visual evidence in Fig. A.4) suggest that women above the ERA or ORA are about 4% points less likely to have had a mammography in the past 12 months.

Table 2 presents the estimated effect of retirement on mammography use and other secondary preventive care outcomes using the bivariate probit model.

**Table 2:** Retirement and secondary preventive care use.

	Mammography use	Manual breast examination	Ovary examination	Pap smear test	Osteoporosis test	Any other gynecological examination
Retired	-0.159***	-0.075*	-0.087***	-0.052	-0.023	-0.079**
	(0.038)	(0.044)	(0.033)	(0.036)	(0.025)	(0.038)
N	17,875	*17,865*	*17,804*	*17,850*	*17,788*	*17,868*

*Sources*: Eurobarometer, own calculations. Estimates are average marginal effects from bivariate probit models. All models include controls for education, country- and year-fixed effects as well as a quadratic age trend. The sample includes women aged 45–75. For all outcomes women were asked whether they had the examination done in the past 12 months. Standard errors are based on 200 bootstrap replications. ***p < 0.01; **p < 0.05; *p < 0.1.

**Fig. 2:**
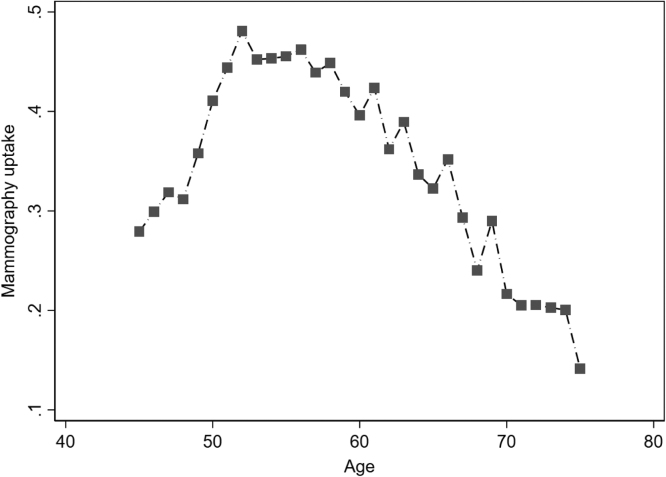
Mammography uptake by age. Source: Eurobarometer, own calculations.

The estimated effects show that being retired reduces the probability of mammography use by about 16% points in the full sample. This is a large reduction in participation. However, we note that mammography use declines by over 20% points between the ages of 50–70 (see Fig. 2) – from almost 50% at age 52 to slightly more than 20% at age 70. Therefore, we argue that a reduction by 16% points is in the plausible range. The reduction in mammography use upon retirement is with about 9% points considerably lower in countries without an organized screening program, and the estimated effect is not statistically significant.

The estimated effects for all other secondary preventive care outcomes are negative as well, although the estimates for pap smear tests and osteoporosis tests are not statistically significant. We also note that the reduction in mammography use is notably larger than the other estimates reported in Table 2.

**Fig. 3:**
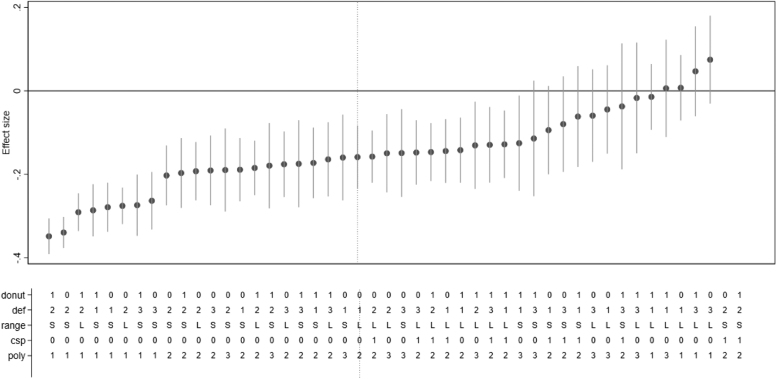
Specification curve for the basic model. Source: Eurobarometer, own calculations. The markers show the point estimates and the lines show 95% confidence intervals for the effect of retirement on mammography use in the past 12 months. The dotted line shows the preferred specification from Table 2. The lower panel shows the model specification. “poly” gives the degree of the polynomial, “csp” indicates whether the age trend is country-specific. “Range” indicates the age range, with “L” standing for ages 45–75, and “S” indicating the age range of the country’s screening program (Table A.1). “Def” gives the definition of retirement status, definition 1 includes homemakers as retired but excludes unemployed women. For definition 2 homemakers are coded as non-retired, and in definition 3 both homemakers and unemployed women are coded as retired. “Donut” indicates whether the first 12 months after the ERA and ORA were excluded or not. All models include further controls for education, and country- and year-fixed effects. Standard errors are based on 200 bootstrap replications. Estimates for the final two model specifications are not shown, because the model did not achieve convergence of the likelihood function.

Fig. 3 shows the specification curve for mammography use. A summary of the different modeling choices is shown in Table A.4 in the Appendix. The estimate from our preferred specification is highlighted by the vertical line. The majority of specifications are negative and significant. There is no clearly discernible pattern among the characteristics of the insignificant specifications. As expected, the specification of the age polynomial is important. However, while all specifications with a quadratic age trend are negative and significant, the proportion of non-significant specification is similar across the other three age trend specifications (4 out of 12 specifications with a linear age trend are insignificant, compared to 5 out of 12 specifications for both country-specific quadratic and cubic age trends).

Table A.5 reports the estimated average marginal effects for robustness checks, in which we only change one aspect at a time. First, we add survey weights to account for non-response. Second, we restrict our sample to the EU15 countries, which are observed in all years. Third, we only use observations of women aged within program age range. Fourth, we divide our sample into a group of countries that had an organized breast cancer screening program in all observed years as well as a group of countries that did not have an organized breast cancer screening program during the study period. We excluded four countries which are observed both before and after the introduction of their organized screening program.^20^ If the timing of the introduction of these screening programs were not exogenous, then the inclusion of these countries could bias our estimates. Fifth, we changed the retirement definition by excluding homemakers, and including unemployed. Sixth, we estimated a “donut” specification where we excluded observations within 12 months of exceeding the ERA or ORA. Finally, we estimated a specification in which we additionally control for marital status and household size.

Overall, these robustness checks confirm our conclusion. We find negative and significant effects of retirement on mammography use in all models. We also conduct a falsification exercise, in which we use placebo state pension ages (Table A.6 in the Online Appendix). We observe no changes in mammography use at these placebo state pension ages in the reduced form regressions, and therefore conclude that these placebo checks support confirm the robustness of our results.^21^ Finally, we compare average marginal effects from our preferred bivariate probit model with estimates from a 2SRI model using logistic regressions in both stages and a linear 2SLS model to check the robustness to functional form assumptions (Table A.7 in the Appendix). The estimated effect of retirement on mammography use is negative and significant in all three models, and the estimated effect in the bivariate probit model is smaller than in the 2SRI and 2SLS models. We therefore conclude that our findings are robust to violations of the required functional form assumptions, and the bivariate probit model can be considered as a “conservative” specification.

## 6 Mechanisms

We investigate whether the effect of retirement on mammography use operates indirectly through changes in income, health status and health knowledge using a mediation analysis.^22^ Table 3 presents the total, direct and indirect (mediating) effects.^23^ Recall that sample sizes are considerably smaller than in the main analysis, and the sample for income may be more selective. We would expect the total effect to be similar to our main analysis (Table 1). However, for income and health there are some differences in the estimated total effects, which limits the external validity of this analysis for our main sample. Nevertheless, we argue that the results can provide suggestive evidence that income, health status and health knowledge are not potential mechanisms of the effect of retirement on mammography use as all indirect effects are very small and not significantly different from zero.

**Table 3:** Potential mechanisms.

	Outcome: Mammography use in the past 12 months		
	A. Health	B. Income	C. Health Knowledge
Total effect	-0.113*	-0.163***	-0.230***
	(0.055)	(0.040)	(0.073)
Direct effect	-0.111*	-0.160***	-0.217***
	(0.056)	(0.040)	(0.074)
Indirect effect	-0.003	-0.003	-0.013
	(0.002)	(0.002)	(0.009)
N	10,498	8736	3807

*Sources*: Eurobarometer, own calculations. Estimates are average marginal effects from a bivariate probit model and a linear 2SLS auxiliary regression model. All models include controls for education, country- and year-fixed effects as well as a quadratic age trend. All models include women aged 45–75. Standard errors are based on 200 bootstrap replications. ***p < 0.001; **p < 0.01; *p < 0.05; † p < 0.1.

## 7 Treatment effect heterogeneity

We re-estimate our bivariate probit model for the subsample of countries with breast cancer screening programs compared to countries without programs, those with low SHI coverage compared to countries with high SHI coverage,^24^ and by educational attainment. The results in Table 4 show that our main result is driven by countries without a program. Moreover, the reduction in mammography use following retirement is considerably larger in countries with lower SHI coverage of healthcare expenditures. This indicates that transitioning out of employment has a larger effect in countries where complementary health insurance covers a smaller share of healthcare expenditures.

**Table 4:** Heterogeneity.

	Outcome: Mammography use in the past 12 months						
	Screening program		Education			SHI coverage	
	No program	Program	Low education	Medium education	High education	Low coverage	High coverage
Retired	-0.165***	-0.093	-0.135**	-0.212***	-0.176**	-0.304***	-0.041
	(0.041)	(0.061)	(0.066)	(0.056)	(0.077)	(0.038)	(0.050)
N	11,313	6562	6796	6823	4209	8154	9419

*Sources*: Eurobarometer, own calculations. Estimates are average marginal effects from bivariate probit models. All models include a quadratic age trend, education and country- and year-fixed effects as well as interaction terms between education and retirement in the second stage and education and the instruments in the first stage. The sample includes women aged 45–75. Standard errors shown in parentheses are based on 200 bootstrap replications. ***p < 0.01; **p < 0.05; *p < 0.1.

Our analysis is based on data covering the period 1996–2006, and therefore does not reflect recent changes of women’s labor force participation. This raises the question whether the estimated effect of retirement on preventive care use is still relevant in more recent years. In the absence of newer data we cannot answer this question with certainty. Instead, we will discuss how recent demographic trends might have affected our estimated effects. This requires careful consideration of whether the estimated effect differs across subgroups of the population. We therefore analyze treatment effect heterogeneity by education. Retirement appears to have a stronger effect on mammography use among medium and high education groups.

## 8 Discussion

We analyze the effect of retirement on mammography use using data from 25 European countries. We address the endogeneity of retirement by using state pension ages for early and official retirement as instruments. Our findings show that retirement reduces mammography use by about 16% points. Retirement also reduces the use of other preventive health check-ups, such as manual breast examinations or ovary examinations. However, the reduction is considerably smaller than the effect of retirement on mammography use. A mammography typically requires an appointment with a radiologist or a specialized mammography unit, while, e.g., manual breast examinations can be conducted during a regular consultation with a GP or gynecologist. This might explain why the latter procedures are less strongly affected by retirement. Nonetheless, our results suggest that the negative effect of retirement is neither specific to mammography nor to breast cancer. Our evidence does not suggest that the reduction in mammography use is driven by changes in health, income, or health knowledge. Instead, we find evidence of important effect heterogeneity, which may be suggestive of specific mechanisms. For instance, organized screening programs might mitigate the negative impact of retirement to some extent, since our estimated effects are significant and robust only in the group of countries without organized screening programs.^25^ However, we note that this heterogeneity by screening program cannot be interpreted as causal, because it may be confounded by unobserved characteristics of countries that introduced screening programs early on. We also find that the negative effect of retirement is stronger in countries with lower SHI coverage of healthcare expenditures, which suggests that access to health care (e.g., due to employer-sponsored complementary health insurance) might partly explain the negative effect. However, we also note that our indicator of healthcare coverage is only a proxy measure, which might also pick up other differences across countries. Our main effect is also stronger among medium and high educated women.

Our results are in line with the health capital model (Galama et al., 2013, Grossman, 1972), which suggests that retirees have fewer incentives to invest in their health and will reallocate some of their health investments into consumption. It appears plausible that this effect would be more pronounced for preventive healthcare (such as cancer screening) than for curative healthcare. While our results are consistent with this hypothesis, we cannot draw any definite conclusions using reduced-form models.

In closing, we acknowledge several limitations of our analysis. The bivariate probit model requires the assumption of joint normality of the error terms. This distributional assumption also contributes to identification in bivariate probit models (Wooldridge, 2010), which means that our estimates might be sensitive to violations of this assumption. However, we also considered a linear 2SLS estimator and a 2SRI model using logistic regression. Both alternative models confirmed that our results are qualitatively similar to those obtained in our bivariate probit model. Moreover, these estimates are even larger in magnitude, and we therefore use the bivariate probit model as a “conservative” specification.

The Eurobarometer data used in this analysis also has several important strengths and limitations. A major strength of the data is that it includes information on secondary preventive care use as well as knowledge of breast cancer prevention and treatment for 25 different European countries. Secondary preventive care use is rarely covered in comparable household surveys. For example, SHARE only includes information on breast cancer screening in wave 2. Moreover, knowledge on cancer is (to the best of our knowledge) not covered in any comparable datasets. Unfortunately, the design of the Eurobarometer as a repeated cross-section means that we cannot examine changes within individuals over time. While panel data methods are not required for the validity of our IV design, it would be very interesting to explore the malleability of cancer knowledge within individuals.

Another limitation is the potential for selective non-response. Response rates for the Eurobarometer are, unfortunately, not routinely published. A report on the 1996 wave of the Eurobarometer (Gallie, 1996) showed considerable variation in the response rate across countries, which suggests that selective non-response might play a role. However, the Eurobarometer data includes post-stratification weights, which are intended to address selective non-response. Our results are not meaningfully altered by the inclusion of these weights for our main sample and the no program sample.

The cross-country setting allows us to examine country-level differences, e.g., in SHI coverage or the existence of a screening program, which are evocative of mechanisms such as price effects. However, there is likely unobserved heterogeneity across countries, including in the strength of the instrument, and we are therefore not able to interpret such country-level differences as causal, nor are we able to directly test the relevant mechanisms due to data limitations. It is left to future research to confirm whether the potential mechanisms suggested by such treatment effect heterogeneity can indeed contribute to our understanding of the negative effect of retirement on mammography use.

It should be noted that the data covers the period of 1996–2006. This is, on the one hand, a very interesting study period due to the variation in pension eligibility ages and breast cancer screening programs between countries as well as within countries over time. However, it also raises the question whether our findings are still relevant today. We argue that this is likely to be the case. First, we have no reason to assume that the underlying mechanisms between retirement and secondary preventive care use have changed substantially over the last decade. However, two important changes that might affect our conclusions are the implementation of breast cancer screening programs in almost all countries studied in this paper, as well as the increase in labor market participation among women.

Following the European Council’s recommendation for population-based breast screening of women aged 50–69 years, almost all countries in the European Union have implemented programs, and existing screening programs have worked towards increasing their participation rates and coverage to reach the target rate of 75%. This might suggest that organized screening programs now are more important in mitigating the negative effect of retirement on breast cancer screening. However, as of 2017 most countries failed to meet the target participation rate of 75% (Ponti et al., 2017), which suggests that socioeconomic characteristics (such as employment) are still relevant determinants of participation.

Finally, labor market participation among older women has become less selective as labor force participation rates have increased in most European countries. This suggests that the composition of women retiring at the pension eligibility ages might have changed. Yet, our analysis of treatment effect heterogeneity shows that the estimated effect of retirement is negative and significant across all levels of educational attainment (but slightly larger for medium and high education). This suggests that our estimated effects should still be relevant in more recent periods. Future research should examine how these changes and trends have affected the relationship between retirement and secondary preventive care use once suitable data becomes available.

## Funding

Peter Eibich acknowledges generous support by the 10.13039/100004440Wellcome Trust (Society & Ethics Research Fellowship 203208/Z/16/Z). The funder was not involved in any aspects of the study.

## Code availability

The statistical code is available from the corresponding author upon request.

## CRediT authorship contribution statement

**Peter Eibich**: Conceptualization, Methodology, Validation, Formal analysis, Data curation, Writing – original draft, Writing – review & editing, Visualization. **Léontine Goldzahl**: Conceptualization, Methodology, Data curation, Writing – original draft, Writing – review & editing, Visualization.

## Conflicts of Interest

The authors declare no competing interests.

## Data Availability

The Eurobarometer data used in this study can be accessed freely via the GESIS Data Service. For further information, please see https://www.gesis.org/eurobarometer-data-service/search-data-access/data-access.

## Footnotes

1 The studies by Hagen (2018) and Hallberg et al. (2015) might differ from other studies, because they focus on earlier (Hallberg et al., 2015) and later retirement (Hagen, 2018), respectively.

2 They report a significant increase for flu shots, i.e., primary prevention.

3 The survey by Nishimura et al. (2018) suggests that the difference in findings can be attributed to methodological differences, and that a unified approach based on state pension ages provides mostly positive or insignificant results. However, there remains considerable heterogeneity, e.g., across countries or health outcomes. For example, while all studies focusing on cognitive functioning find negative effects of retirement (Bingley and Martinello, 2013, Bonsang et al., 2012, Mazzonna and Peracchi, 2016, Mazzonna and Peracchi, 2012, Rohwedder and Willis, 2010), studies focusing on mortality mostly find a reduction in mortality (Bloemen et al., 2017, Bozio et al., 2021, Hallberg et al., 2015) or no significant effects (Hagen, 2018, Hernaes et al., 2013) with two studies reporting an increase in mortality after retirement (Fitzpatrick and Moore, 2018, Kuhn et al., 2020).

4 These prices were collected in 2020 such that these prices may have been different at the time of the survey.

5 There is an ongoing debate about how the benefits of mammography (i.e., reduced mortality) should be weighed against the cost associated with false positive results and overdiagnosis possibly leading to overtreatment (Gøtzsche and Jørgensen, 2013). A U.K. expert panel conducted a meta-analysis of the randomized trial evidence and concluded that screening mammograms reduced the relative risk of mortality by 20%, while acknowledging the problems of overdiagnosis and false-positive biopsies (Marmot et al., 2013).

6 For instance, if eligible women are invited every 2 years, a screening uptake rate of 70% corresponds to the ratio between the number of women screened and the number of eligible women divided by two.

7 Retirement at the ORA might also involve a minimum period of social security contributions. In contrast, early retirement at the ERA might be limited to certain occupations or sectors. In addition, schemes offering an ERA might also involve a trade-off between an earlier retirement entry and a reduced pension.

8 In detail, these are the EB44.3 (1996), EB47.2 (1997), EB49 (1998), EB59 (2003) and EB66.2 (2006). Questions on secondary preventive care for women were only introduced in these five waves of the Eurobarometer survey.

9 This does not completely solve the issue of selective labor market participation. In particular, our sample might include women who permanently left the labor market after having children, and thus will not have done any paid work for a number of years. Unfortunately, we do not have any further information, e.g., when the respondent left their previous job.

10 The exact wording is “Do you personally think that cancer can be prevented, or not?” Available answers (one answer allowed) were “No, it cannot be prevented” “Yes, it can be prevented by a better balanced diet and by leading a healthy lifestyle” “Yes, it can be prevented by teaching people to recognize the signs of cancer” “Yes, it can be prevented by regular medical check-ups” “Yes, it can be prevented by other means” “Don’t Know”. The item takes 1 if the respondent answers “Yes”, and 0 otherwise.

11 There are no data on Malta and Cyprus.

12 The ERA varies between 52 and 65, while the ORA varies between 55 and 67.

13 Table B.1 in the Online Appendix provides summary statistics with separate means for countries with and without screening programs.

14 Fig. A.2 shows the same plot separately for each country.

15 We also estimated a reduced form regression of our instruments on marital status and household size. As expected, the estimated effects are not significant and relatively small in magnitude (between −1.7% points and +1.8% points). The results are available on request.

16 These assumptions are: *(i)* The instrument should be correlated with the treatment, *(ii)* the instrument should not affect the outcome except through its effect on the treatment, *(iii)* the instrument should have a monotone effect on the treatment. We assess assumption *(i)* visually and statistically. We argue that assumption *(ii)* should hold conditionally on age. For assumption *(iii)*, we argue that it is highly implausible that reaching age threshold for pension eligibility would reduce a women’s likelihood to retire.

17 This implies that we also directly include retirement in the second stage of the model, rather than predicted retirement based on the first stage.

18 We examined the distribution of the bootstrapped standard errors for different numbers of replications by re-estimating our preferred specification 100 times. The results (available upon request) show that the distribution for 200 replications is very similar to the distribution for 500 replications.

19 Stratifying the sample is more flexible than interacting the retirement variable with the categories of interest, because it does not assume that age trends are similar across categories.

20 These countries are Belgium, France, the Netherlands, and Portugal. While Hungary, Lithuania and Cyprus introduced their screening program during the study period, we only observe these countries in 2006 (i.e., after the introduction of their program).

21 The bivariate probit model shows a significant negative effect even when using placebo state pension ages. As we explain in more detail in the Online Appendix (Table A.6), this effect comes from the identification based on functional form assumptions in the bivariate probit model, and therefore does not threaten the validity of our results.

22 It should be noted that this analysis does not account for endogeneity of health, income or health knowledge as potential mediators. This might introduce some bias, and thus the results cannot be interpreted as causal. Nevertheless, we argue that these findings provide suggestive evidence against these mediators. For example, even though we find a positive (causal) effect of retirement on good health, and a negative association between good health and screening participation, the estimated effects are too small to account for a meaningful proportion of the negative effect of retirement on mammography use.

23 In the Appendix, Tables A.8 and A.9 shows the results of the effect of retirement on health status, income and health knowledge. Retirement has an impact on income, but not on health or health knowledge. However, we note that the results for health are sensitive to the choice of the cut-off used in dichotomizing self-assessed health. If we define “good health” as a self-assessed health of “good” or “very good” compared to “fair”, “bad” or “very bad”, then we find a significant positive effect of retirement on health.

24 The sample with lower SHI coverage includes Estonia, Spain, Finland, Greece, Hungary, Lithuania, Latvia, Poland, Portugal, Slovenia and Slovakia in all years. The sample with higher SHI coverage includes Czech Republic, Germany, Denmark, France, UK, Luxembourg and Sweden in all years. The remaining countries (Austria, Belgium, Ireland, Italy and The Netherlands) change groups across the years. We also conducted several robustness checks, e.g., using only EU15 countries, excluding countries who change groups from the analysis, or using average SHI coverage to construct a classification with stable groups across the years. The results confirm that our findings are highly robust and are available on request.

25 In Online Appendix B, we reproduce the most important figures and tables separately for countries with and those without a screening program.
