# Supplementary material for: Does retirement affect secondary preventive care use? Evidence from breast cancer screening
Source: Econ Hum Biol. 2021 Dec;43:101061. doi: 10.1016/j.ehb.2021.101061 (PMC8683749; doi:10.1016/j.ehb.2021.101061)
Supplement: Supplementary material [file mmc1.docx]

**Online appendix**

1. **Supplementary Figures and Tables**

**Table A1: Retirement ages and screening programs in Europe**

|  | **Retirement ages of European women** | | | | | | | | | | **Breast cancer Screening program characteristics** | |
| --- | --- | --- | --- | --- | --- | --- | --- | --- | --- | --- | --- | --- |
| **Survey years** | 1996 | | 1997 | | 1998 | | 2003 | | 2006 | |  |  |
|  | ERA | ORA | ERA | ORA | ERA | ORA | ERA | ORA | ERA | ORA | Year of implementation  (nationwide coverage) | Age range |
| 1. **Screening program introduced before observed period (pre-1996)** | | | | | | | | | | | | |
| Finland | 58 | 65 | 58 | 65 | 60 | 65 | 60 | 65 | 62 | 65 | 1989 | 50-69 |
| Luxembourg | 60 | 65 | 60 | 65 | 60 | 65 | 60 | 65 | 60 | 65 | 1992 | 50-69 |
| UK | 60 | 60 | 60 | 60 | 60 | 60 | 60 | 60 | 60 | 60 | 1995 | 50-70^b^ |
| Sweden | 60 | 65 | 60 | 65 | 61 | 65 | 61 | 65 | 61 | 65 | 1996^a^ | 40-74 |
| 1. **Screening program introduced during observed period (1996-2006)** | | | | | | | | | | | | |
| Netherlands | 60 | 65 | 60 | 65 | 60 | 65 | 65 | 65 | 62 | 65 | 1997 ^a^ | 50-74^c^ |
| Belgium | 60 | 60 | 60 | 61 | 60 | 61 | 60 | 63 | 60 | 64 | 2001 | 50-69 |
| Hungary |  |  |  |  |  |  |  |  | 57 | 62 | 2002 | 45-65 |
| France | 55 | 60 | 55 | 60 | 55 | 60 | 55 | 60 | 55 | 60 | 2004 ^a^ | 50-74 |
| Lithuania |  |  |  |  |  |  |  |  | 55 | 60 | 2005 | 50-69 |
| Portugal | 60 | 62 | 60 | 62 | 60 | 64.5 | 55 | 65 | 55 | 65 | 2005 ^a^ | 45-69 |
| Cyprus |  |  |  |  |  |  |  |  | 63 | 65 | 2006 ^a^ | 50-69 |
| 1. **Screening program introduced after observed period (post-2006)/no existing screening program** | | | | | | | | | | | | |
| Czech republic |  |  |  |  |  |  |  |  | 56.5 | 59.8 | 2007 ^a^ | 45-69 |
| Estonia |  |  |  |  |  |  |  |  | 56.5 | 59.5 | 2007 ^a^ | 50-65 |
| Italy^d^ | 52 | 57 | 52 | 57 | 54 | 58 | 57 | 60 | 57 | 60 | 2007 ^a^ | 50-69 |
| Poland |  |  |  |  |  |  |  |  | 55 | 60 | 2007 ^a^ | 50-69 |
| Austria | 55 | 60 | 55 | 60 | 55 | 60 | 56.5 | 60 | 57 | 60 | 2008 ^a^ | 40-69 |
| Ireland | 65 | 65 | 65 | 65 | 65 | 65 | 65 | 65 | 65 | 65 | 2008 | 50-64 |
| Slovenia |  |  |  |  |  |  |  |  | 58 | 61 | 2008 | 50-69 |
| Germany | 60 | 65 | 60 | 65 | 60 | 65 | 60 | 65 | 60 | 65 | 2009 ^a^ | 50-69 |
| Latvia |  |  |  |  |  |  |  |  | 59 | 61 | 2009 | 50-69 |
| Malta |  |  |  |  |  |  |  |  | 60 | 60 | 2009 | 50-59 |
| Spain | 61 | 65 | 61 | 65 | 61 | 65 | 61 | 65 | 61 | 65 | 2009 | 50-69 |
| Denmark | 60 | 67 | 60 | 67 | 60 | 67 | 60 | 67 | 60 | 65 | 2010 | 50-69 |
| Slovakia |  |  |  |  |  |  |  |  | 62 | 62 | 2019 | 50-69 |
| Greece | 55 | 60 | 55 | 60 | 55 | 60 | 55 | 60 | 55 | 60 | None | - |

Notes: Empty cells mean that our dataset does not contain observations for the country in that specified year. Source: Social protection in the member states of the European Union, MISSOC (1994, 1997, 1998, 2003), Celidoni and Rebba (2017), Euwals et al.(2010), Jousten et al.(2010), Mazzonna and Peracchi (2014) and Staubli and Zweimuller (2013) for the retirement ages and Altobelli and Lattanzi (2014) for the program characteristics.

^a^ indicates that regional program existed before it reached nationwide coverage.

^b^ In the dataset we distinguish between Great Britain and Northern Ireland. In Northern Ireland, the age range of the program was 50-64 until 2003, and 50-70 from 2004 onwards.

^c^ The age range was 50-70 in 1997, and 50-74 from 1998 onwards. ERA and ORA are respectively the early retirement age and official retirement age.

^d^ The ERA for Italy refers to the eligibility age for the so-called “seniority pension”, which is only available for individuals who have worked for at least 35 years.

| **Table A.2: Number of observations per country and year** | | | | | | | |
| --- | --- | --- | --- | --- | --- | --- | --- |
| **Country/Year** | | **1996** | **1997** | **1998** | **2003** | **2006** | ***Total*** |
| Austria |  | 190 | 181 | 186 | 223 | 221 | ***1,001*** |
| Belgium |  | 113 | 116 | 146 | 163 | 219 | ***757*** |
| Cyprus |  | 0 | 0 | 0 | 0 | 186 | ***186*** |
| Czech Republic | | 0 | 0 | 0 | 0 | 321 | ***321*** |
| Denmark |  | 195 | 198 | 205 | 228 | 233 | ***1,059*** |
| Estonia |  | 0 | 0 | 0 | 0 | 365 | ***365*** |
| Finland |  | 225 | 221 | 197 | 270 | 275 | ***1,188*** |
| France |  | 186 | 157 | 161 | 166 | 204 | ***874*** |
| Germany |  | 442 | 440 | 445 | 525 | 375 | ***2,227*** |
| Greece |  | 142 | 167 | 157 | 143 | 182 | ***791*** |
| Hungary |  | 0 | 0 | 0 | 0 | 333 | ***333*** |
| Ireland |  | 161 | 124 | 155 | 145 | 204 | ***789*** |
| Italy |  | 185 | 140 | 135 | 180 | 168 | ***808*** |
| Latvia |  | 0 | 0 | 0 | 0 | 295 | ***295*** |
| Lithuania |  | 0 | 0 | 0 | 0 | 325 | ***325*** |
| Luxembourg | | 75 | 91 | 70 | 96 | 119 | ***451*** |
| Malta |  | 0 | 0 | 0 | 0 | 116 | ***116*** |
| Netherlands | | 163 | 168 | 162 | 202 | 280 | ***975*** |
| Poland |  | 0 | 0 | 0 | 0 | 249 | ***249*** |
| Portugal |  | 204 | 208 | 190 | 214 | 246 | ***1,062*** |
| Slovakia |  | 0 | 0 | 0 | 0 | 351 | ***351*** |
| Slovenia |  | 0 | 0 | 0 | 0 | 283 | ***283*** |
| Spain |  | 145 | 103 | 89 | 113 | 184 | ***634*** |
| Sweden |  | 232 | 211 | 204 | 228 | 263 | ***1,138*** |
| UK |  | 225 | 216 | 250 | 268 | 338 | ***1,297*** |
| ***Total*** |  | ***2,883*** | ***2,741*** | ***2,752*** | ***3,164*** | ***6,335*** | ***17,875*** |
| Source: Eurobarometer, own calculations. The sample includes women aged 45 to 75, for whom information on retirement status and age is available. | | | | | | | |

| **Table A.3: Summary statistics** | | | | | | | | |
| --- | --- | --- | --- | --- | --- | --- | --- | --- |
| **Variable** | **Mean** | **SD** | **Min** | **Max** | **N** | **Mean retired** | **Mean working** | |
| *Preventive care use* | | | | | | | | |
| Mammography in the past 12 months | 0.364 | 0.481 | 0 | 1 | 17,875 | 0.335 | 0.400 | *** |
| Manual breast examination in the past 12 months | 0.402 | 0.490 | 0 | 1 | 17,865 | 0.359 | 0.458 | *** |
| Ovary examination in the past 12 months | 0.231 | 0.422 | 0 | 1 | 17,804 | 0.188 | 0.285 | *** |
| Pap smear test in the past 12 months | 0.346 | 0.476 | 0 | 1 | 17,850 | 0.276 | 0.435 | *** |
| Osteoporosis test in the past 12 months | 0.158 | 0.365 | 0 | 1 | 17,788 | 0.179 | 0.131 | *** |
| Any other gynecological examination in the past 12 months | 0.322 | 0.467 | 0 | 1 | 17,868 | 0.266 | 0.392 | *** |
| *Perceptions of early detection, prevention and treatment of breast cancer (1997 and 1998 only)* | | | | | | | | |
| Agreed: The sooner a cancer is detected, the better it can be treated. | 0.979 | 0.143 | 0 | 1 | 5,347 | 0.978 | 0.980 |  |
| Agreed: A mammography will detect signs of breast cancer. | 0.964 | 0.187 | 0 | 1 | 5,217 | 0.967 | 0.959 |  |
| Agreed: There are effective treatments for breast cancer. | 0.896 | 0.305 | 0 | 1 | 4,744 | 0.894 | 0.898 |  |
| Agreed: In most cases, you can be cured of breast cancer if it is detected early enough. | 0.937 | 0.242 | 0 | 1 | 5,030 | 0.935 | 0.940 |  |
| Agreed: Removal of the breast is the only way to be cured of breast cancer. | 0.232 | 0.422 | 0 | 1 | 4,626 | 0.266 | 0.190 | *** |
| Agreed: Do you personally think that cancer cannot be prevented? | 0.405 | 0.491 | 0 | 1 | 5,108 | 0.429 | 0.376 | *** |
| *Covariates* | | | | | | | | |
| Country offers organized screening program | 0.367 | 0.482 | 0 | 1 | 17,875 | 0.351 | 0.388 | *** |
| Retired | 0.560 | 0.496 | 0 | 1 | 17,875 | 1 | 0 |  |
| …of which homemakers | 0.147 | 0.354 | 0 | 1 | 17,875 | 0.262 | 0 |  |
| Age | 58.396 | 8.748 | 45 | 75 | 17,875 | 62.956 | 52.597 | *** |
| *Survey year* | | | | | | | | |
| Year 1996 |  |  |  |  | 2,883 |  |  |  |
| Year 1997 | |  |  |  | 2,741 |  |  |  |
| Year 1998 | |  |  |  | 2,752 |  |  |  |
| Year 2003 | |  |  |  | 3,164 |  |  |  |
| Year 2006 | |  |  |  | 6,335 |  |  |  |
| *Age when finished full-time education* | | | | | | | | |
| 15 or younger | 0.380 | 0.485 | 0 | 1 | 17,875 | 0.475 | 0.259 | *** |
| 16 -19 | 0.382 | 0.486 | 0 | 1 | 17,875 | 0.354 | 0.417 | *** |
| 20 and above | 0.235 | 0.424 | 0 | 1 | 17,875 | 0.171 | 0.318 | *** |
| Still studying | 0.003 | 0.051 | 0 | 1 | 17,875 | 0.000 | 0.006 | *** |
| Sources: EB66.2, EB59.0, EB49, EB47.2, EB44.3. Notes: The last column shows the significance of a t-test | | | | | | | | |
| for equality of means between working and non-working women. *** p<0.01, ** p<0.05, * p<0.1. | | | | | | | | |

**
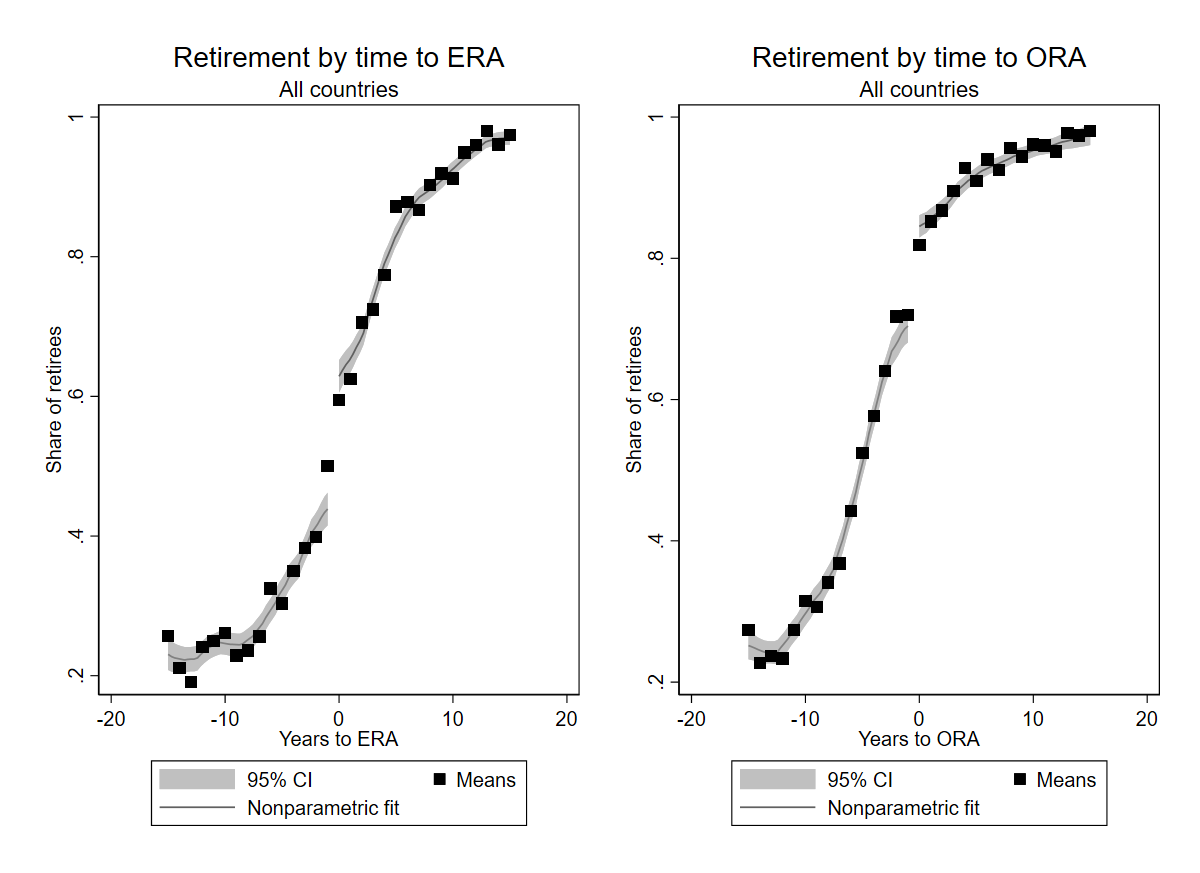
**

**Fig. A.1** Share of retirees by time to state pension age. Source: Eurobarometer, own calculation. “Years to ERA” and “Years to ORA” are calculated by subtracting age from the relevant ERA or ORA. The markers show average retirement rates against year to ERA/ORA. The lines show local polynomial fits on both sides of the threshold, and the gray areas show 95 percent confidence intervals around the fit.


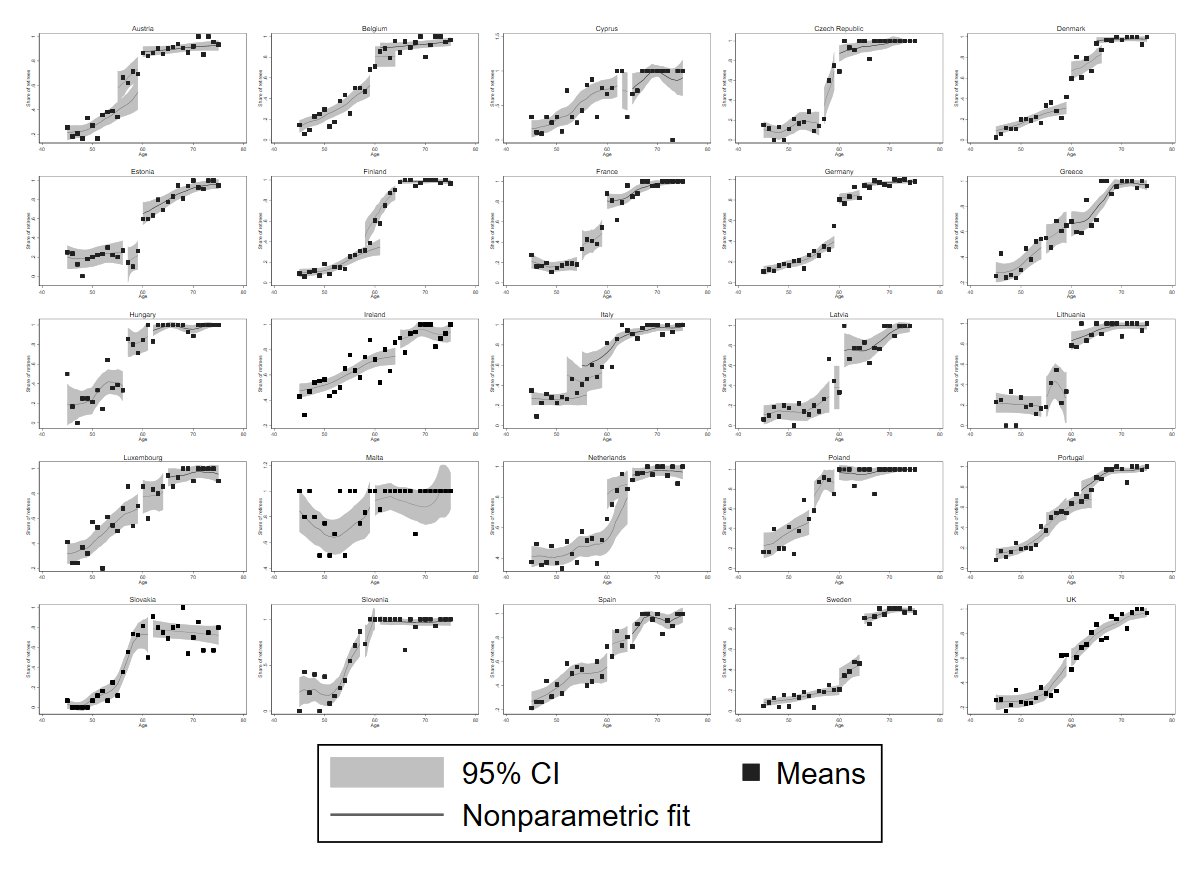


**Fig. A.2** Share of retirees by age for individual countries. Source: Eurobarometer, own calculation. The markers show average retirement rates against age. The lines show local polynomial fits for women aged below their country’s ERA, those aged between the ERA and the ORA, and those aged above their country’s ORA, respectively. The gray areas show 95 percent confidence intervals around the fit. Due to pension reforms, it is possible that in some countries women of the same age are assigned to different groups based on the year of the survey, in which case the fitted lines may overlap.


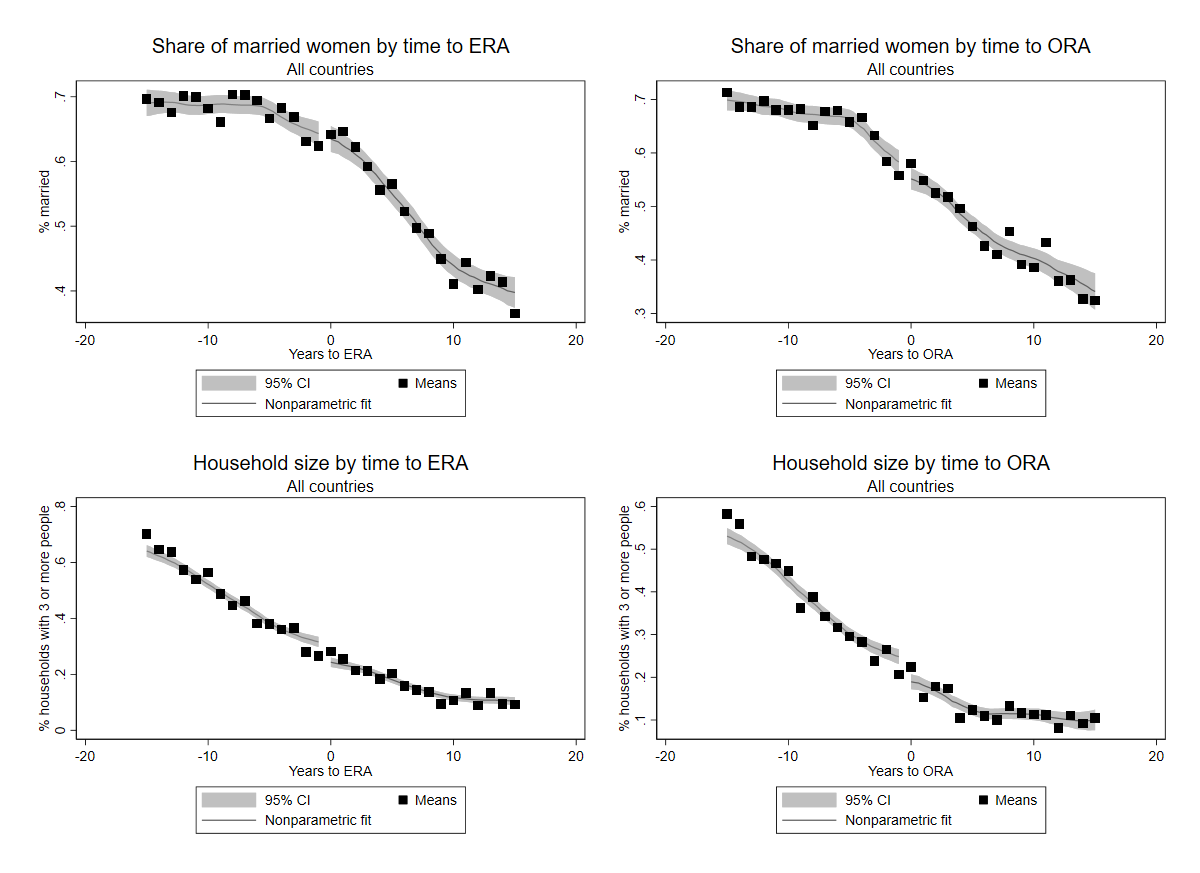


**Fig. A.3** Marital status and household size by age Source: Eurobarometer, own calculations. The markers show means and the lines are local polynomial fits. Years to ERA and Years to ORA are calculated by subtracting the ERA or ORA from the women’s age.


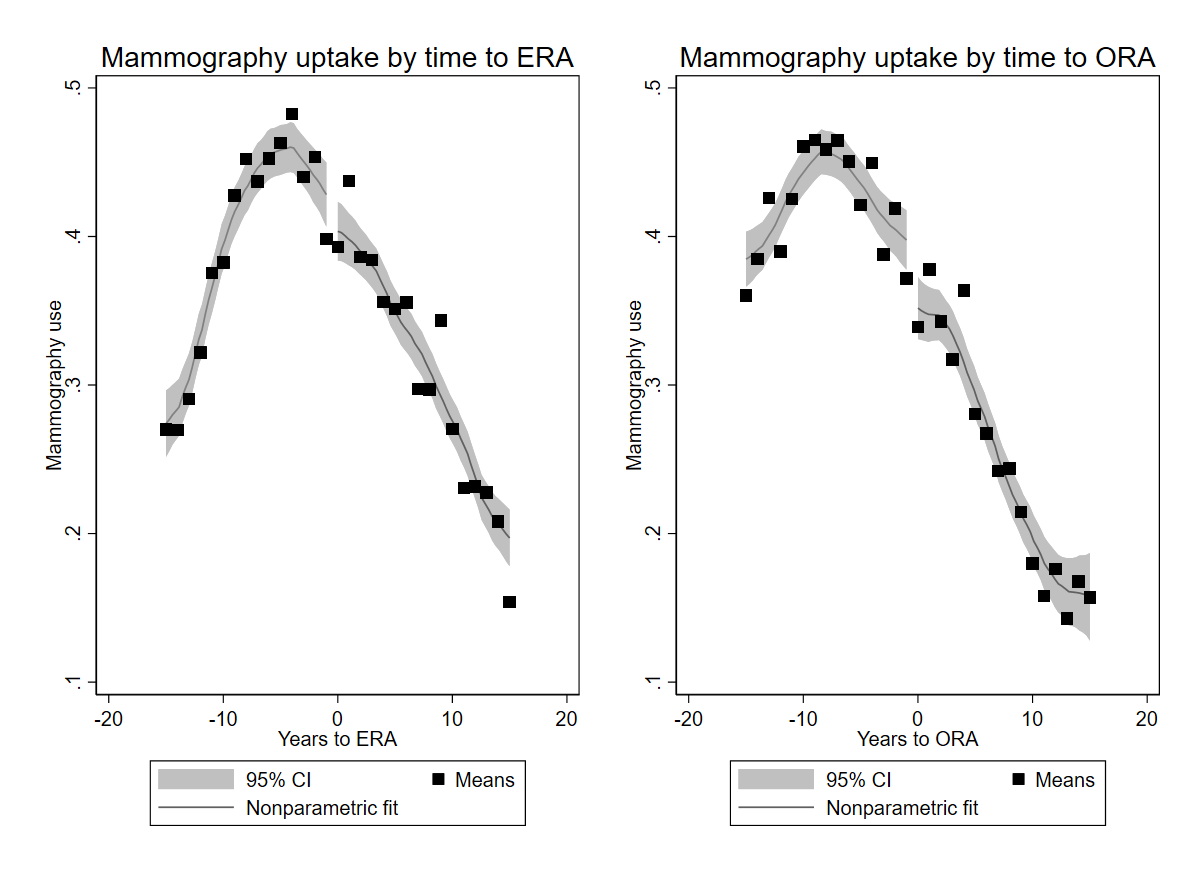


**Fig. A.4** Mammography uptake by time to state pension age. Source: Eurobarometer, own calculation. “Years to ERA” and “Years to ORA” are calculated by subtracting age from the relevant ERA or ORA. The markers show average mammography use rates against year to ERA/ORA. The lines show local polynomial fits on both sides of the threshold, and the gray areas show 95 percent confidence intervals around the fit.

**
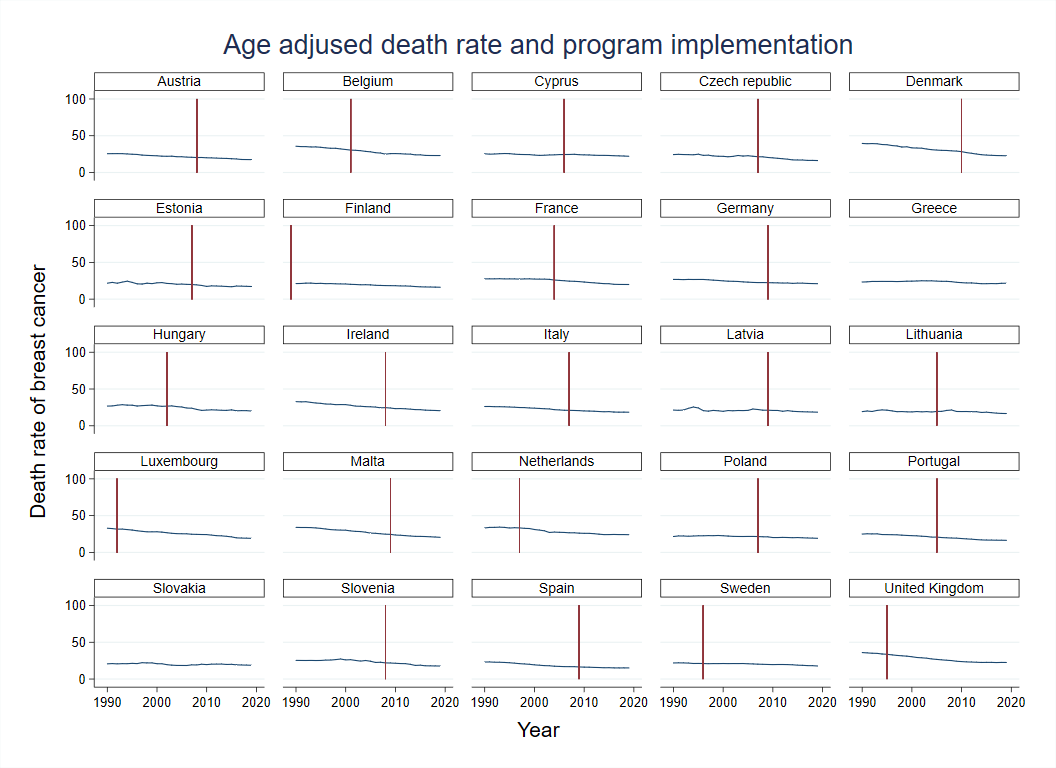
**

**Fig. A.5** Age-adjusted death rate between 1980 and 2019 by country. Source: Global Burden of Disease Collective Network (2021) . Red line indicates year of nationwide program implementation.

**Table A.4 Summary of characteristics shown in the specification curve**

| Specifications |  |
| --- | --- |
| Donut | 0: full sample;  1: omitting observations within the first 12 months after passing the ERA/ORA. |
| Retirement definition | 0: including homemakers as retired and excluding unemployed women as working;  1: excluding homemakers as working;  2: including unemployed women as retired. |
| Age range | L: full sample aged between 45 and 75;  S: within the age range of their country’s screening program. |
| Country specific age trend | 0: No, common age trend for the full sample;  1: Yes, age trend interacted with country dummy. |
| Polynomial for the age trend | 1: first degree – linear trend;  2: second degree – quadratic trend;  3: third degree -cubic trend. |

| **Table A.5: Robustness checks** | | | | | | | | | | | | | | | | | |
| --- | --- | --- | --- | --- | --- | --- | --- | --- | --- | --- | --- | --- | --- | --- | --- | --- | --- |
|  | **Original model** |  | **Survey weights** |  | **Only EU15 countries** |  | **Within program age range^a^** |  | **Without changes in program existence** |  | **Retirement excludes homemakers** |  | **Retirement includes unemployed** |  | **Excluding observations <12months after ERA/ORA** |  | **Controls for household size and marital status** |
| **Retired** | -0.159*** |  | -0.168*** |  | -0.160*** |  | -0.163*** |  | -0.128*** |  | -0.192*** |  | -0.176*** |  | -0.142*** |  | -0.150*** |
|  | (0.037) |  | (0.038) |  | (0.038) |  | (0.041) |  | (0.040) |  | (0.032) |  | (0.040) |  | (0.038) |  | (0.044) |
|  |  |  |  |  |  |  |  |  |  |  |  |  |  |  |  |  |  |
| N | *17,875* |  | *17,875* |  | *15,051* |  | *13,285* |  | *14,207* |  | *17,875* |  | *17,875* |  | *16,850* |  | *14,626* |
| ^a^ The model includes only observations within the age range of the respective program. For countries without a mammography program, we only used observations between age 50 and age 69. | | | | | | | | | | | | | | | | | |
| ^b^ This result is only driven by Finland because it has a program in all years. Excluding Finland yield to a non-significant effect of retirement. | | | | | | | | | | | | | | | | | |
| Sources: Eurobarometer, own calculations. All models include controls for education, country- and year-fixed effects as well as a quadratic age trend. All models except the one in column 3 and 4 include women aged 45 to 75. Standard errors are based on 200 bootstrap replications. The model using survey weights uses robust standard errors. *** p<0.01; ** p<0.05; * p<0.1. | | | | | | | | | | | | | | | | | |

| **Table A.6: Placebo state pension ages** | | | | | | | | | |
| --- | --- | --- | --- | --- | --- | --- | --- | --- | --- |
| *Outcome: Mammography use in the past 12 months* | | | | | | | | | |
|  | **Bivariate probit** | | | |  | **2SLS estimator** | | | |
|  | *Main specification* | | *Placebo SPA* | |  | *Main specification* | | *Placebo SPA* | |
| *IV estimates* | | | | | | | | | |
| **Retired** | -0.159 | *** | 0-.129 | *** |  | -0.318 | *** | -0.243 |  |
|  | (0.036) |  | (0.048) |  |  | (0.054) |  | (0.216) |  |
| *First stage estimates* | | | | | | | | | |
| **Above ERA** | 0.107 | *** | 0.008 |  |  | 0.198 | *** | 0.029 | *** |
|  | (0.010) |  | (0.008) |  |  | (0.013) |  | (0.010) |  |
| **Above ORA** | 0.055 | *** | 0.021 | *** |  | 0.090 | *** | 0.041 | *** |
|  | (0.013) |  | (0.007) |  |  | (0.011) |  | (0.009) |  |
| *Reduced form estimates* | | | | | | | | | |
| **Above ERA** | -0.044 | *** | -0.003 |  |  | -0.050 | *** | -0.006 |  |
|  | (0.013) |  | (0.010) |  |  | (0.015) |  | (0.011) |  |
| **Above ORA** | -0.039 | *** | -0.008 |  |  | -0.050 | *** | -0.011 |  |
|  | (0.014) |  | (0.009) |  |  | (0.014) |  | (0.010) |  |
| Source: Eurobarometer, own calculations. Columns 1 and 3 (“Main specification”) show estimates using actual state pension ages as instruments. Columns 2 and 4 (“Placebo SPA”) show estimates using placebo state pension ages as instruments. Placebo state pension ages were generated by randomly drawing a number between 50 and 70 from a uniform distribution for each observation. All models include controls for education, country- and year-fixed effects as well as a quadratic age trend. The sample includes women aged 45-75. Standard errors are based on 200 bootstrap replications. *** p<0.01; ** p<0.05; * p<0.1. | | | | | | | | | |

The results shown in Table A.6 above show that when using placebo state pension ages, estimated effects in the reduced form regression are very close to zero and not statistically significant. Likewise, estimates of the first stage regression show that the instruments are considerably smaller, and the ERA is not statistically significant in the bivariate probit model. However, identification in the bivariate probit model comes both from the exclusion restriction on the excluded instruments, as well as from functional form assumptions on the joint distribution of the error terms in the first and second stage of the model. If these functional form assumptions hold, then we could in principle identify the effect of retirement on mammography use without an excluded instrument. Placebo state pension ages are essentially irrelevant explanatory variables in the first stage regression, and thus the model with placebo state pension ages should be very similar to a model without excluded instruments. Thus, the negative and significant effect of retirement on mammography use in the bivariate probit model with placebo state pension ages is likely driven by these functional form assumptions. We argue that this does not threaten the validity of our results, because *(i)* the estimated effects in the first stage and reduced form regression are very close to zero, and *(ii)* Table A.7 shows that our results are robust to different functional form assumptions,

| **Table A.7: Robustness to functional form assumptions** | | | | | |  |
| --- | --- | --- | --- | --- | --- | --- |
|  | *Outcome: Mammography use in the past 12 months* | | | | |  |
|  | **Bivariate probit model** |  | **2SLS model** |  | **2SRI (Logit-Logit)** |  |
| **Retired** | -0.159*** |  | -0.318*** |  | -0.200*** |  |
|  | (0.036) |  | (0.055) |  | (0.047) |  |
|  |  |  |  |  |  |  |
|  |  |  |  |  |  |  |
| N | 17,875 |  | *17,875* |  | *17,875* |  |
| Sources: Eurobarometer, own calculations. All models include controls for education, country- and year-fixed effects as well as a quadratic age trend. The sample includes women aged 45-75. Standard errors are based on 200 bootstrap replications. *** p<0.01; ** p<0.05; * p<0.1. | | | | | |  |
|  |  |  |  |  |  |  |
|  |  |  |  |  |  |  |
|  |  |  |  |  |  |  |

| **Table A.8: Retirement and cancer knowledge** | | | | | | | | | | | |
| --- | --- | --- | --- | --- | --- | --- | --- | --- | --- | --- | --- |
|  | **The sooner a cancer is detected, the better it can be treated.** |  | **A mammography will detect signs of breast cancer.** |  | **There are effective treatments for breast cancer.** |  | **In most cases, you can be cured of breast cancer if it is detected early enough.** |  | **Removal of the breast is the only way to be cured of breast cancer.** |  | **Do you personally think that cancer cannot be prevented?** |
| **Retired** | -0.056 |  | 0.032 |  | -0.120*** |  | 0.048 |  | 0.044 |  | 0.202** |
|  | (0.041) |  | (0.031) |  | (0.046) |  | (0.037) |  | (0.059) |  | (0.079) |
|  |  |  |  |  |  |  |  |  |  |  |  |
| *Mean* | *0.979* |  | *0.964* |  | *0.896* |  | *0.937* |  | *0.232* |  | *0.405* |
|  |  |  |  |  |  |  |  |  |  |  |  |
| N | *5,346* |  | *5,216* |  | *4,743* |  | *5,029* |  | *4,625* |  | *5,107* |
| Sources: Eurobarometer, own calculations. All models include controls for education, country-and-year fixed effects as well as quadratic age trend. The sample includes women aged 45-75. The outcome variables are binary indicators showing whether respondents agreed or disagreed with the statement. Standard errors are based on 200 bootstrap replications. *** p<0.01; ** p<0.05; * p<0.1. | | | | | | | | | | | |
|  |  |  |  |  |  |  |  |  |  |  |  |
|  |  |  |  |  |  |  |  |  |  |  |  |

| **Table A.9: Retirement and potential mediators** | | | | | |  |
| --- | --- | --- | --- | --- | --- | --- |
|  | **Good self-reported health** |  | **Household income above median** |  | **Health Knowledge Index** |  |
| **Retired** | -0.011 |  | -0.119** |  | -0.176 |  |
|  | (0.024) |  | (0.049) |  | (0.330) |  |
|  |  |  |  |  |  |  |
| *N* | *10,585* |  | *8,777* |  | *3,807* |  |
| Sources: Eurobarometer, own calculations. All models include controls for education, country-and-year fixed effects as well as quadratic age trend. The sample includes women aged 45-75. “Good self-reported health” is a binary variable for the three highest categories of the 5-point self-reported health measure. “Household income above median” refers to the country-specific median income. The “Health Knowledge Index” is derived by summing up across all measures of health knowledge. It was only available in year 1997 and 1998. Estimates for good self-reported health and household income above median are average marginal effects from a bivariate probit model. Estimates for the Health Knowledge Index come from a linear two-stage least squares model. Standard errors for the bivariate probit model are based on 200 bootstrap replications, and for the linear 2SLS model robust standard errors are shown. *** p<0.01; ** p<0.05; * p<0.1. | | | | | |  |
|  |  |  |  |  |  |  |
|  |  |  |  |  |  |  |

**B. Results for countries with and without screening programs**

| **Table B.1: Summary statistics** | | | | | | | | |
| --- | --- | --- | --- | --- | --- | --- | --- | --- |
| **Variable** | **Mean** | **SD** | **Min** | **Max** | **N** | **Mean Program** | **Mean No Program** | |
| *Preventive care use* | | | | | | | | |
| Mammography in the past 12 months | 0.364 | 0.481 | 0 | 1 | 17,875 | 0.431 | 0.325 | *** |
| Manual breast examination in the past 12 months | 0.402 | 0.490 | 0 | 1 | 17,865 | 0.390 | 0.409 | ** |
| Ovary examination in the past 12 months | 0.231 | 0.422 | 0 | 1 | 17,804 | 0.186 | 0.257 | *** |
| Pap smear test in the past 12 months | 0.346 | 0.476 | 0 | 1 | 17,850 | 0.329 | 0.355 | *** |
| Osteoporosis test in the past 12 months | 0.158 | 0.365 | 0 | 1 | 17,788 | 0.126 | 0.177 | *** |
| Any other gynecological examination in the past 12 months | 0.322 | 0.467 | 0 | 1 | 17,868 | 0.259 | 0.358 | *** |
| *Perceptions of early detection, prevention and treatment of breast cancer (1997 and 1998 only)* | | | | | | | | |
| Agreed: The sooner a cancer is detected, the better it can be treated. | 0.979 | 0.143 | 0 | 1 | 5,347 | 0.991 | 0.974 | *** |
| Agreed: A mammography will detect signs of breast cancer. | 0.964 | 0.187 | 0 | 1 | 5,217 | 0.966 | 0.963 |  |
| Agreed: There are effective treatments for breast cancer. | 0.896 | 0.305 | 0 | 1 | 4,744 | 0.966 | 0.863 | *** |
| Agreed: In most cases, you can be cured of breast cancer if it is detected early enough. | 0.937 | 0.242 | 0 | 1 | 5,030 | 0.962 | 0.926 | *** |
| Agreed: Removal of the breast is the only way to be cured of breast cancer. | 0.232 | 0.422 | 0 | 1 | 4,626 | 0.153 | 0.270 | *** |
| Agreed: Do you personally think that cancer cannot be prevented? | 0.405 | 0.491 | 0 | 1 | 5,108 | 0.503 | 0.364 | *** |
| *Covariates* | | | | | | | | |
| Country offers organized screening program | 0.367 | 0.482 | 0 | 1 | 17,875 | 1 | 0 |  |
| Retired | 0.560 | 0.496 | 0 | 1 | 17,875 | 0.535 | 0.574 | *** |
| …of which homemakers | 0.147 | 0.354 | 0 | 1 | 17,875 | 0.134 | 0.154 | *** |
| Age | 58.396 | 8.748 | 45 | 75 | 17,875 | 58.351 | 58.423 |  |
| *Age when finished full-time education* | | | | | | | | |
| 15 or younger | 0.380 | 0.485 | 0 | 1 | 17,875 | 0.345 | 0.401 | *** |
| 16 -19 | 0.382 | 0.486 | 0 | 1 | 17,875 | 0.377 | 0.384 |  |
| 20 and above | 0.235 | 0.424 | 0 | 1 | 17,875 | 0.275 | 0.213 | *** |
| Still studying | 0.003 | 0.051 | 0 | 1 | 17,875 | 0.003 | 0.002 |  |
| Sources: EB66.2, EB59.0, EB49, EB47.2, EB44.3. Notes: The last column shows the significance of a t-test | | | | | | | | |
| for equality of means between working and non-working women. *** p<0.01, ** p<0.05, * p<0.1. | | | | | | | | |


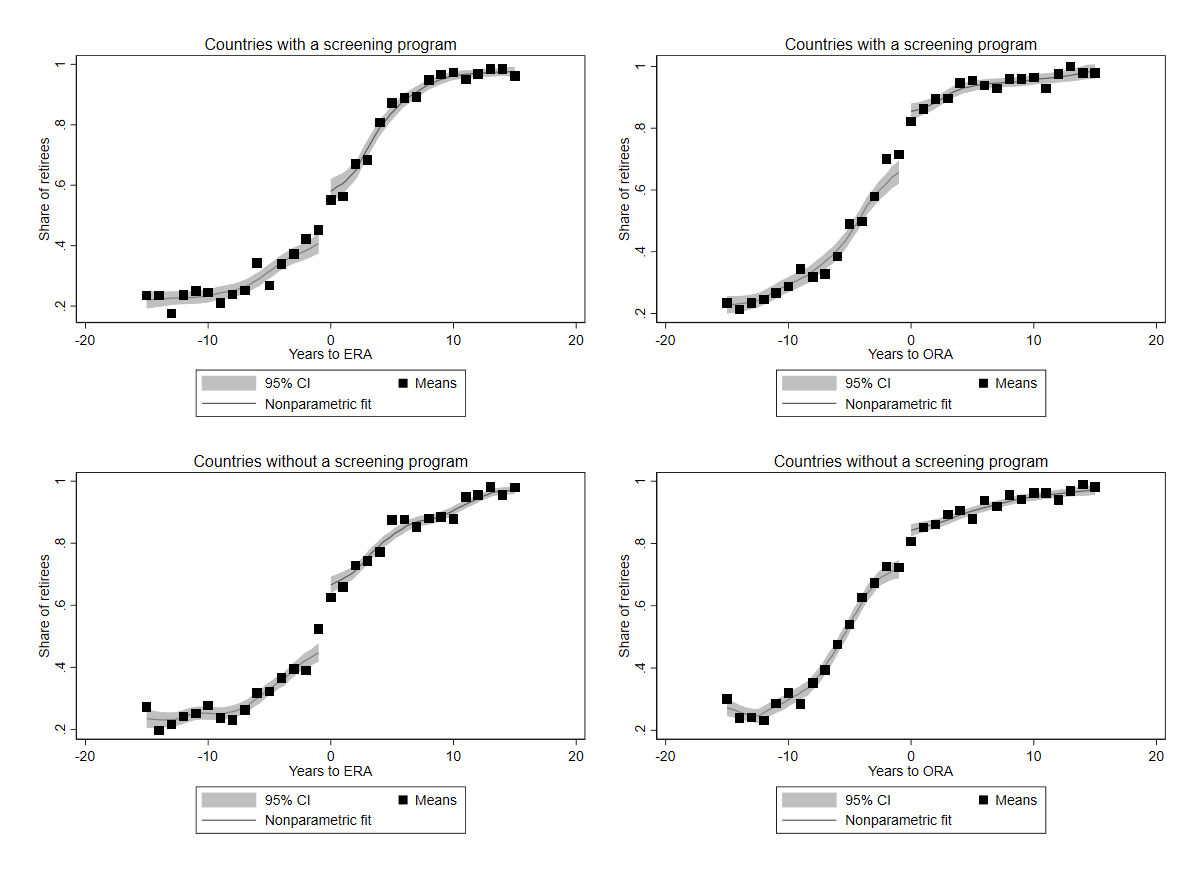
 **Fig. B.1** Retirement by time to state pension age. Source: Eurobarometer, own calculation. “Years to ERA” and “Years to ORA” are calculated by subtracting age from the relevant ERA or ORA. The markers show average mammography use rates against year to ERA/ORA. The lines show local polynomial fits on both sides of the threshold, and the gray areas show 95 percent confidence intervals around the fit.


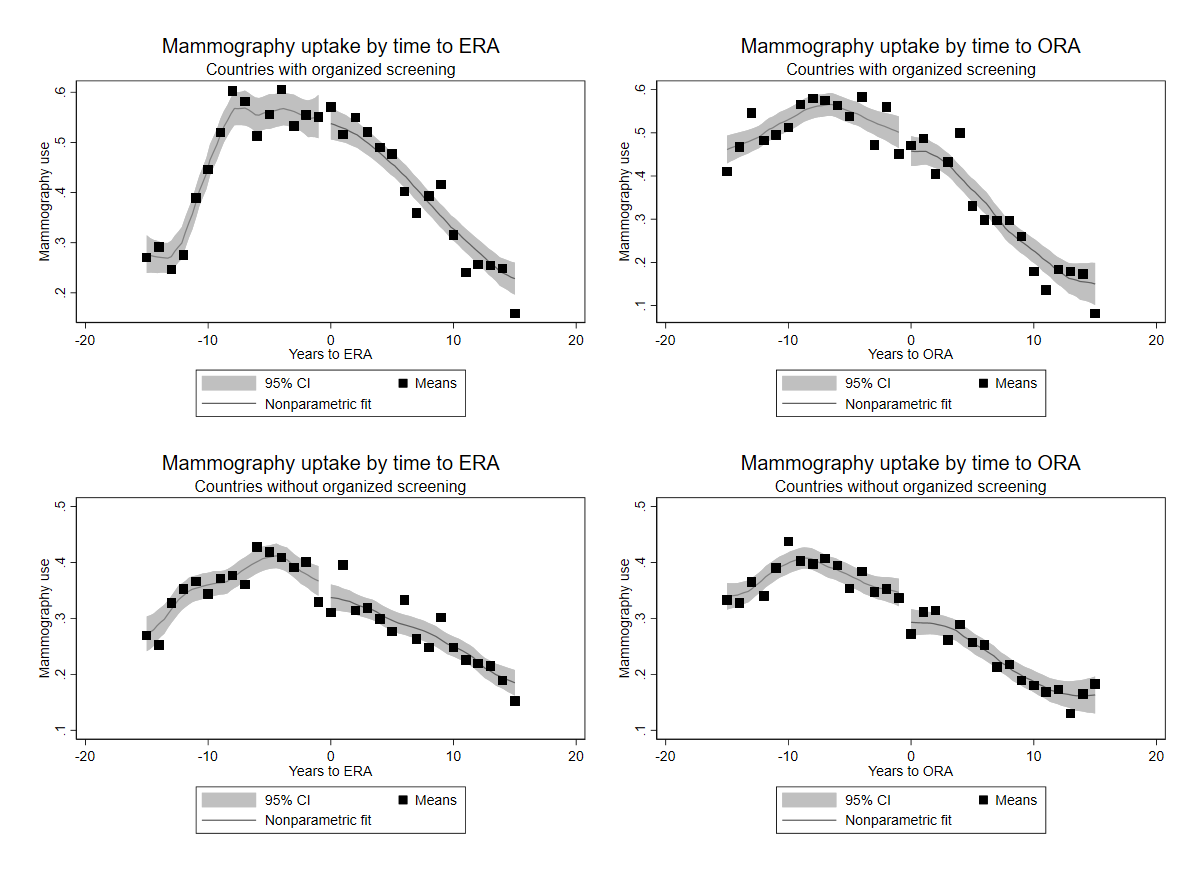
**Fig. B.2** Mammography uptake by time to state pension age. Source: Eurobarometer, own calculation. “Years to ERA” and “Years to ORA” are calculated by subtracting age from the relevant ERA or ORA. The markers show average mammography use rates against year to ERA/ORA. The lines show local polynomial fits on both sides of the threshold, and the gray areas show 95 percent confidence intervals around the fit.

| **Table B.2: First-stage regression and reduced form estimates** | | | | | |  |
| --- | --- | --- | --- | --- | --- | --- |
|  | *First stage* | |  | *Reduced form* | |  |
|  | *No program* | *Program* |  | *No program* | *Program* |  |
| **Above ERA** | 0.116*** | 0.083*** |  | -0.044*** | -0.040 |  |
|  | (0.014) | (0.018) |  | (0.017) | (0.025) |  |
| **Above ORA** | 0.046*** | 0.066*** |  | -0.044** | -0.006 |  |
|  | (0.017) | (0.023) |  | (0.019) | (0.026) |  |
|  |  |  |  |  |  |  |
| *N* | *11,313* | *6,562* |  | *11,313* | *6,562* |  |
| Sources: Eurobarometer, own calculations. All models include a country-specific quadratic age trend, education and country-by-year fixed effects. The interacted model includes a control variable for program existence and age range of the program. The sample includes women aged 45-75. Standard errors shown in parentheses are two-way clustered on country-year and age in the first-stage model, and robust standard errors in the reduced form. *** p<0.01; ** p<0.05; * p<0.1. | | | | | |  |
|  |  |  |  |  |  |  |
|  |  |  |  |  |  |  |

| **Table B.3: Mediation analysis** | | | | | | | | |
| --- | --- | --- | --- | --- | --- | --- | --- | --- |
|  | *A. Health* | |  | *B. Income* | |  | *C. Health Knowledge* | |
|  | *No program* | *Program* |  | *No program* | *Program* |  | *No program* | *Program* |
| **Total Effect** | -0.178*** | -0.009 |  | -0.091 | -0.152 |  | -0.168* | -0.244 |
|  | (0.061) | (0.096) |  | (0.062) | (0.097) |  | (0.093) | (0.205) |
| **Direct Effect** | -0.176*** | -0.006 |  | -0.086 | -0.153 |  | -0.154* | -0.251 |
|  | (0.061) | (0.096) |  | (0.062) | (0.097) |  | (0.092) | (0.206) |
| **Indirect Effect** | -0.002 | -0.004 |  | -0.004 | 0.001 |  | -0.013 | 0.006 |
|  | (0.003) | (0.006) |  | (0.004) | (0.004) |  | (0.016) | (0.012) |
| N | 6,407 | 4,091 |  | 5,789 | 2,947 |  | 2,502 | 1,305 |
| Sources: Eurobarometer, own calculations. All models include controls for education, country- and year-fixed effects as well as a quadratic age trend. All models include women aged 45 to 75. Standard errors are based on 200 bootstrap replications. *** p<0.001; ** p<0.01; * p<0.05; † p<0.1. | | | | | | | | |

| **Table B.4: Retirement and potential mediators** | | | | | |  |
| --- | --- | --- | --- | --- | --- | --- |
|  | **Good self-reported health** |  | **Household income above median** |  | **Health Knowledge Index** |  |
| *A. No program* | | | | | |  |
| **Retired** | 0.023 |  | -.198*** |  | 0.373 |  |
|  | (0.032) |  | (0.058) |  | (0.464) |  |
|  |  |  |  |  |  |  |
| *N* | *6,459* |  | *5,814* |  | *2502* |  |
| *B. Program* | | | | | |  |
| **Retired** | -0.059* |  | 0.091 |  | -1.32** |  |
|  | (0.033) |  | (0.089) |  | (0.570) |  |
|  |  |  |  |  |  |  |
| *N* | *4,126* |  | *2,963* |  | *1,305* |  |
| Sources: Eurobarometer, own calculations. All models include controls for education, country-and-year fixed effects as well as quadratic age trend. The sample includes women aged 45-75. “Good self-reported health” is a binary variable for the two highest categories of the 5-point self-reported health measure. “Household income above median” refers to the country-specific median income. The “Health Knowledge Index” is derived by summing up across all measures of health knowledge. It was only available in year 1997 and 1998. Estimates for good self-reported health and household income above median are average marginal effects from a bivariate probit model. Estimates for the Health Knowledge Index come from a linear two-stage least squares model. Standard errors for the bivariate probit model are based on 200 bootstrap replications, and for the linear 2SLS model robust standard errors are shown. *** p<0.01; ** p<0.05; * p<0.1. | | | | | |  |
|  |  |  |  |  |  |  |
|  |  |  |  |  |  |  |

| **Table B.5: Heterogeneity** | | | | | | |  |
| --- | --- | --- | --- | --- | --- | --- | --- |
|  | **Education** | | |  | **SHI Coverage** | |  |
|  | *Low education* | *Medium education* | *High education* |  | *Low coverage* | *High coverage* |  |
| *A. No program* | | | | | | |  |
| **Retired** | -0.134 | -0.182** | -0.242*** |  | -0.309*** | -0.070 |  |
|  | (0.082) | (0.075) | (0.083) |  | (0.052) | (0.048) |  |
|  |  |  |  |  |  |  |  |
| N | 4,533 | 4,349 | 2,405 |  | 5,367 | 5,830 |  |
| *B. Program* | | | | | | |  |
| **Retired** | -0.239*** | -0.208** | 0.023 |  | -0.260*** | 0.084 |  |
|  | (0.087) | (0.098) | (0.138) |  | (0.061) | (0.095) |  |
|  |  |  |  |  |  |  |  |
| N | 2,263 | 2,474 | 1,804 |  | 2,787 | 3,589 |  |
| Sources: Eurobarometer, own calculations. Estimates shown are average marginal effects. All models include a quadratic age trend, education and country- and year-fixed effects as well as interaction terms between education and retirement in the second stage and education and the instruments in the first stage. The sample includes women aged 45-75. Standard errors shown in parentheses are based on 200 bootstrap replications. *** p<0.01; ** p<0.05; * p<0.1. | | | | | | |  |
|  |  |  |  |  |  |  |  |
|  |  |  |  |  |  |  |  |
|  |  |  |  |  |  |  |  |

| **Table B.6: Robustness checks** | | | | | | | | | | | | | | | | | |
| --- | --- | --- | --- | --- | --- | --- | --- | --- | --- | --- | --- | --- | --- | --- | --- | --- | --- |
|  | **Original model** |  | **Survey weights** |  | **Only EU15 countries** |  | **Within program age range^a^** |  | **Without changes in program existence** |  | **Retirement excludes homemakers** |  | **Retirement includes unemployed** |  | **Excluding observations <12months after ERA/ORA** |  | **Controls for household size and marital status** |
| *A. No program* | | | | | | | | | | | | | | | | | |
| **Retired** | -0.165*** |  | -0.145*** |  | -0.154*** |  | -0.168*** |  | -0.130** |  | -0.170*** |  | -0.216*** |  | -0.136*** |  | -0.175*** |
|  | (0.044 |  | (0.049) |  | (0.050) |  | (0.052) |  | (0.051) |  | (0.041) |  | (0.048) |  | (0.049) |  | (0.049) |
|  |  |  |  |  |  |  |  |  |  |  |  |  |  |  |  |  |  |
| N | *11,313* |  | *11,313* |  | *9,333* |  | *8,225* |  | *9,289* |  | *11,313* |  | *11,313* |  | *10,657* |  | *9,317* |
| *B. Program* | | | | | | | | | | | | | | | | | |
| **Retired** | -0.093 |  | -0.155** |  | -0.096 |  | -0.049 |  | -0.059 |  | -0.148*** |  | -0.069 |  | -0.099 |  | -0.078 |
|  | (0.064) |  | **^b^** (0.066) |  | (0.066) |  | (0.083) |  | (0.068) |  | (0.056) |  | (0.070) |  | (0.072) |  | (0.076) |
|  |  |  |  |  |  |  |  |  |  |  |  |  |  |  |  |  |  |
| N | *6,562* |  | *6,562* |  | *5,718* |  | *5,060* |  | *4,918* |  | *6,562* |  | *6,562* |  | *6,193* |  | *5,309* |
| ^a^ The model includes only observations within the age range of the respective program. For countries without a mammography program, we only used observations between age 50 and age 69. | | | | | | | | | | | | | | | | | |
| ^b^ This result is only driven by Finland because it has a program in all years. Excluding Finland yield to a non-significant effect of retirement. | | | | | | | | | | | | | | | | | |
| Sources: Eurobarometer, own calculations. All models include controls for education, country- and year-fixed effects as well as a quadratic age trend. All models except the one in column 3 and 4 include women aged 45 to 75. Standard errors are based on 200 bootstrap replications. The model using survey weights uses robust standard errors. *** p<0.01; ** p<0.05; * p<0.1. | | | | | | | | | | | | | | | | | |

1. **Countries without a screening program**
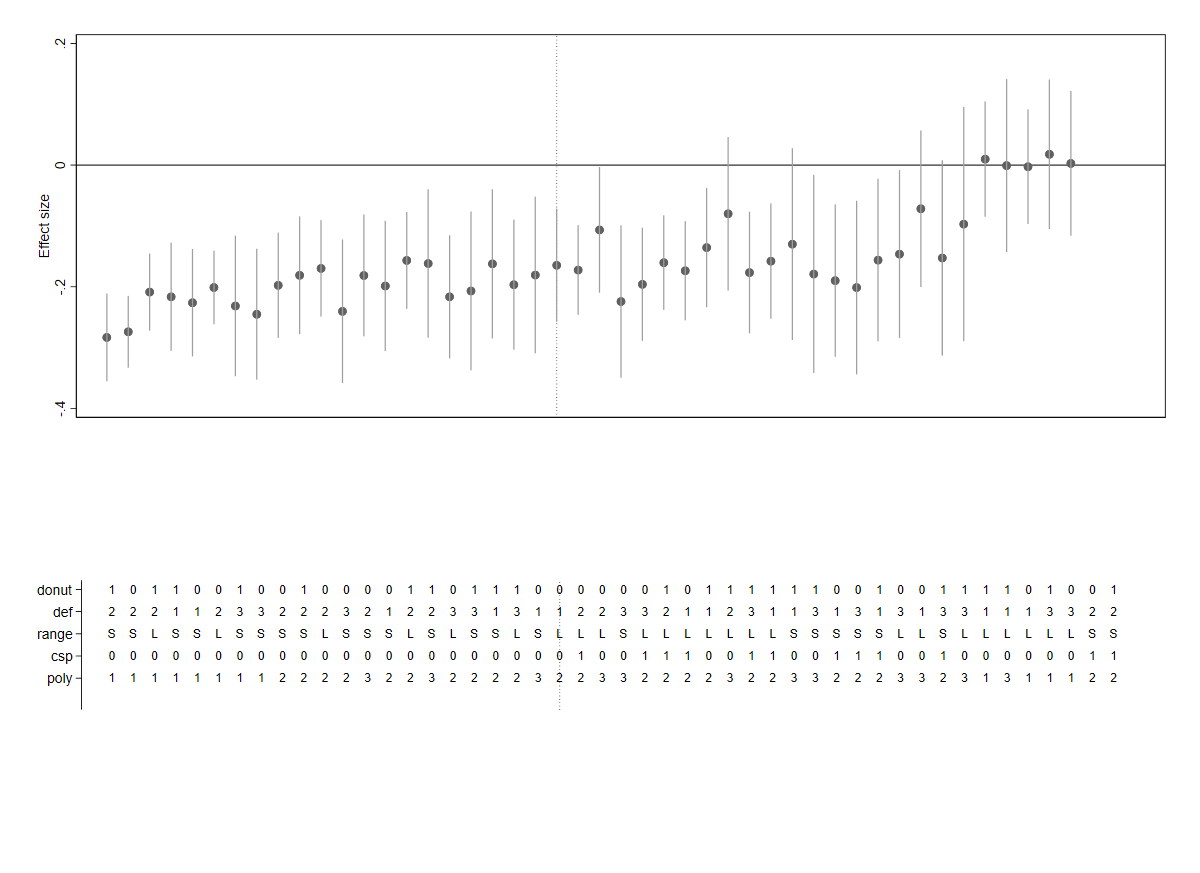

2. **Countries with a screening program**


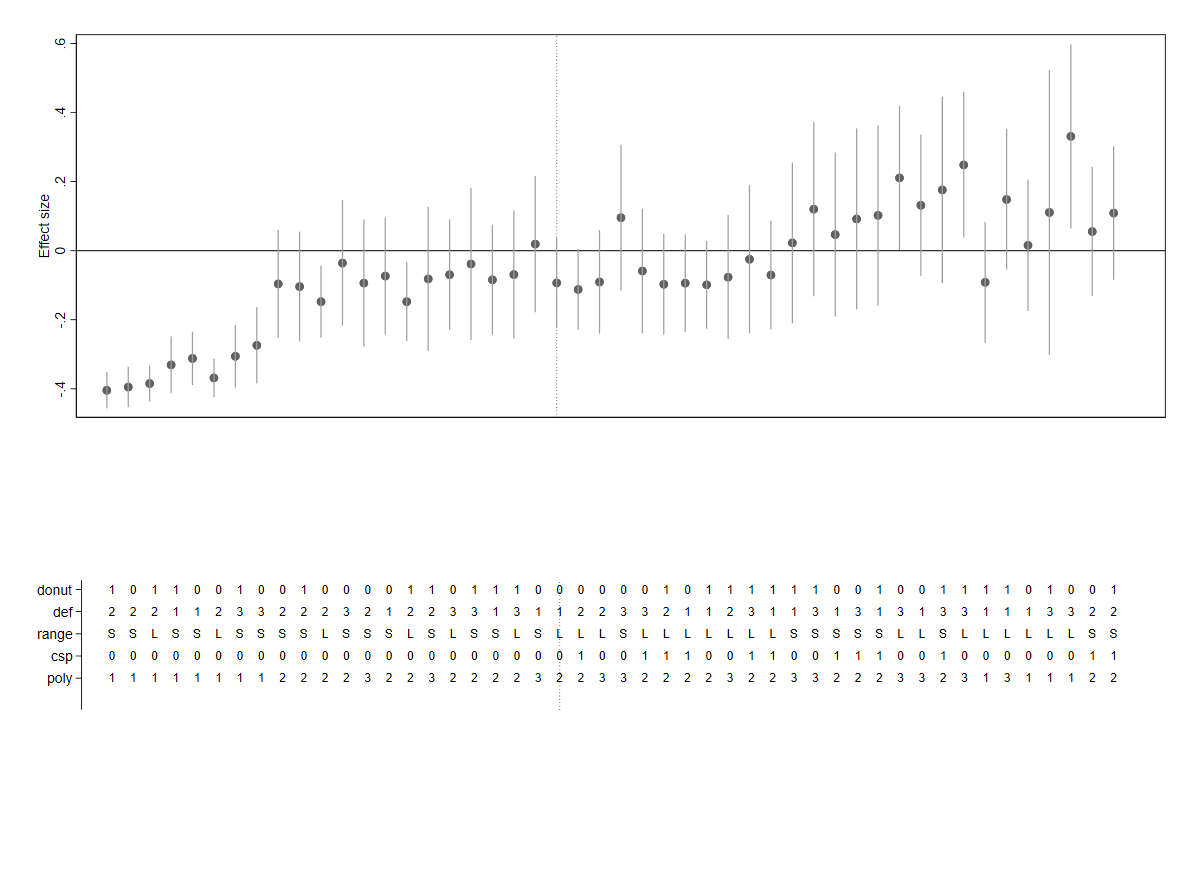


**Fig. B.3** Specification curves for countries with and without screening programs. Source: Eurobarometer, own calculations. Panel A shows the effect of retirement in countries without an organized screening program, and panel b shows the effect of retirement in countries with an organized screening program. The markers show the average marginal effects from a bivariate probit model and the lines show 95 percent confidence intervals for the effect of retirement on mammography use in the past 12 months. The dotted line shows the preferred specification from Table 2. The lower panel shows the model specification. “poly” gives the degree of the polynomial, “csp” indicates whether the age trend is country-specific. “Range” indicates the age range, with “L” standing for ages 45-75, and “S” indicating the age range of the country’s screening program (Table A.1). “Def” gives the definition of retirement status, definition 1 includes homemakers as retired but excludes unemployed women. For definition 2 homemakers are coded as non-retired, and in definition 3 both homemakers and unemployed women are coded as retired. “Donut” indicates whether the first 12 months after the ERA and ORA were excluded or not. All models include further controls for education, and country- and year-fixed effects. Standard errors are based on 200 bootstrap replications. Panel A excludes estimates for the final two model specifications, because the model did not achieve convergence of the likelihood function.

**References**

Altobelli, E., & Lattanzi, A. (2014). Breast cancer in European Union: An update of screening programmes as of March 2014. *International Journal of Oncology*, *45*(5), 1785–1792.

Celidoni, M., & Rebba, V. (2017). Healthier lifestyles after retirement in Europe? Evidence from SHARE. *The European Journal of Health Economics*, *18*(7), 805–830. https://doi.org/10.1007/s10198-016-0828-8

Euwals, R., van Vuuren, D., & Wolthoff, R. (2010). Early Retirement Behaviour in the Netherlands: Evidence From a Policy Reform. *De Economist*, *158*(3), 209–236. https://doi.org/10.1007/s10645-010-9139-0

Global Burden of Disease Collective Network. (2021). *Global Burden of Disease Study 2019: Reference Life Table*. INstitute for Health Metrics and Evaluation (IHME).

Jousten, A., Lefèbvre, M., Perelman, S., & Pestieau, P. (2010). The Effects of Early Retirement on Youth Unemployment: The Case of Belgium. In *Social Security Programs and Retirement around the World: The Relationship to Youth Employment* (pp. 47–76). University of Chicago Press. http://www.nber.org/chapters/c8251

Mazzonna, F., & Peracchi, F. (2014). *Unhealthy retirement? Evidence of occupation heterogeneity*. USI Università della Svizzera italiana. https://ideas.repec.org/p/lug/wpidep/1401.html

Staubli, S., & Zweimüller, J. (2013). Does raising the early retirement age increase employment of older workers? *Journal of Public Economics*, *108*(Supplement C), 17–32. https://doi.org/10.1016/j.jpubeco.2013.09.003
